# C–C Bond Formation during Electrochemical CO_2_ Reduction on Pristine Cu(100) Unlikely to Involve Adsorbed CO at Any Potential

**DOI:** 10.1021/jacs.5c20300

**Published:** 2026-02-12

**Authors:** John Mark P. Martirez, Emily A. Carter

**Affiliations:** † Princeton Plasma Physics Laboratory, Princeton, New Jersey 08543-0451, United States; ‡ Department of Mechanical and Aerospace Engineering and the Andlinger Center for Energy and the Environment, Princeton University, Princeton, New Jersey 08544-5263, United States

## Abstract

Formation of hydrocarbons containing two or more carbon atoms (C_2+_) during heterogeneous electrochemical CO and CO_2_ reduction (ECOR and ECO_2_R) only occurs, among pure metals, on Cu electrodes. Moreover, the activity and selectivity is facet dependent, with Cu(100) generally preferentially forming ethylene over methane. Previously, we found via quantum-mechanics-based modeling that, unlike standard density functional theory, more accurate correlated wavefunction methods predict that non-electroactive coupling pathways involving two adsorbed COs (*CO) or a *CO and a *COH to form C–C bonds on Cu(100) are kinetically inhibited, with the former also thermodynamically unfavorable. Here, we extend that embedded complete active space second order perturbation theory (ECASPT2) study, further showing that electrochemical coupling of two *COs to form an anionic dimer [OC*–*CO]^(1+δ)–^, followed by protonation to form [OC*–*COH]^δ−^, is not kinetically competitive with the reduction of *CO to *COH at relevant ECO/CO_2_R potentials. Our simulations therefore suggest that the ability of Cu(100) to electrochemically synthesize C_2+_ molecules from CO and CO_2_ is unlikely to be via *CO, at least on pristine Cu(100). Instead, hydrogenated CO species (*COH, *CH_*x*_OH, or *CH_*x*_) are most likely to be the key intermediates in C–C bond formation.

## Graphical abstract

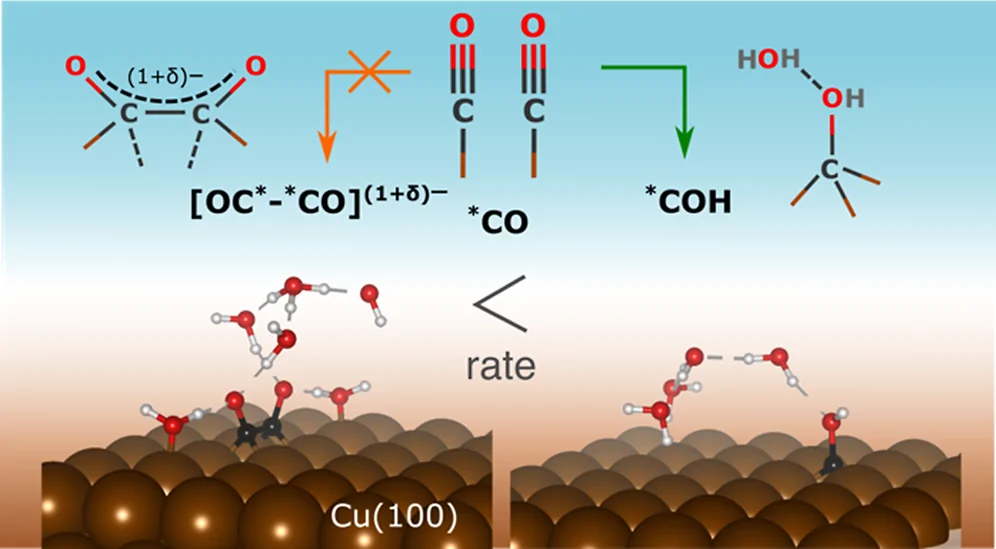

## Introduction

Understanding electrochemical CO and CO_2_ reduction (ECOR and ECO_2_R) to hydrocarbons on Cu electrodes, which are among the few known heterogeneous catalysts for these reactions thus far,^1^ will help bring CO_2_ conversion to useful products to reality. Even on Cu, ECOR and ECO_2_R suffer from low hydrocarbon yield and selectivity where in most cases hydrogen is preferentially produced.^1–4^ Current understanding of ECO/CO_2_R on Cu has focused on adsorbed CO, *CO, (* means adsorbed; the adjacent element indicates the point of attachment to the surface) as a central surface species involved in crucial elementary steps limiting the rate and selectivity of hydrocarbon formation.^5–16^ Notably, crystal facet dependence of selectivity^3,5–7,17^ between methane and ethylene is yet to be fully understood and the definitive role of *CO in the branching of product profiles remains in question. For example, Cu(111) favors formation of methane over ethylene, but the reverse is true for Cu(100).^4,6^ Reduction of CO/CO_2_ to hydrocarbons and oxygenates requires partial or full hydrogenation of C and may involve C–O bond scission and C–C bond formation. Naturally, the competition between hydrogenation of *CO to hydroxymethylidyne (*COH) or formyl (*CHO) and coupling of two *COs to form a C–C bond has been studied extensively,^8–16^ because these pathways can lead to divergence in product outcomes, where the former may favor C_1_ products, such as methane and methanol, whereas the latter may favor C_2_ products, such as ethylene and ethanol. One may wonder why the field has been driven to focus on the early steps as being crucial in product selectivity. This possibly is due to a series of density functional theory (DFT)-based simulations that almost universally conclude that *CO, *COH, and *CHO can couple with little to no barrier on Cu(100).^8–16^ Therefore, although without direct proof, it is also believed in some experimental literature that the emergence of C_1_ and C_2_ products must be controlled by the competition between hydrogenation and coupling of the three aforementioned species.^5–8^ The work by Perez-Gallent et al. claimed direct proof of the formation of OC*–*COH during ECOR on Cu(100) using Fourier transform infrared (FTIR) spectroscopy, from what the authors interpret as signatures of C=O and C–OH stretches of OC*–*COH after consulting DFT-predicted normal modes of *CO, *COH, and *CHO coupling products.^18^ Arguably, the work’s interpretation was self-prophesying in that they assume that neither *COH and *CHO could have formed within the potential range they conducted the measurements, and the authors really only considered for evaluation and spectroscopic assignments the dimer O*C*–CO and, because it can be subsequently hydrogenated or lithiated, OC*–*COH, OC*–*CHO, and OC*–*COLi.^18^ More recently, Kim et al. showed, using time-resolved attenuated total-reflection surface-enhanced infrared absorption spectroscopy, that direct *CO coupling primarily occurs at grain boundaries on a highly defective sprayed powder Cu.^19^ Further experimental and theoretical studies are needed to identify the relevant C_2_ intermediate(s), even on just the pristine low-index facets.

DFT has cemented its role as an invaluable companion to experiments in demystifying the complexity of ECO/CO_2_R catalysis at a fundamental level. However, the approximation to its solution, i.e., the coupled one-electron equations approach and the single-electron configuration representation within the Kohn–Sham formalism, and exchange–correlation (XC) functional approximations, in many cases lead to both qualitative and quantitative errors, namely, underestimation of bond dissociation barriers and too strong adsorption, artificial charge transfer or overdelocalization (due to nonvanishing electron self-interaction), and incorrect relative phase stability of solids and adsorption preferences for molecules.^20–24^ Many DFT XC functionals, for example, incorrectly predict preferential binding of *CO to hollow sites^25–28^ as opposed to the experimentally observed atop sites^29,30^ on Cu. This error is due to artificially enhanced π-back bonding from the metal to the molecule, especially amplified at high coordination number sites, e.g., hollow sites, a consequence of charge overdelocalization and possibly a too-low acceptor π* CO energy. Sharifzadeh et al. and Zhao et al. showed that using, e.g., complete active space second order perturbation theory (CASPT2)^31–33^ within the embedded correlated wavefunction (ECW) formalism together with density functional embedding theory (DFET),^34–36^ the correct site preference for CO on Cu(111) and Cu(100) emerges.^37–39^ In ref 38, conversion of *CO to *COH was predicted by embedded CASPT2 (ECASPT2) to be kinetically dominant over production of *CHO, via proton-coupled electron transfer (PCET) on Cu(100), where the free energy barrier is ≤0.52 eV at electrode potentials ≤ −0.4 V vs the reversible hydrogen electrode (RHE). In a companion paper to the present work we show, also using ECASPT2, that non-electroactive couplings of *CO and *COH to OC*–*CO and OC*–*COH have insurmountable free energy barriers of 1.7 and 1.2 eV, respectively.^40^ Further, while only a small barrier (0.27 eV) is found to form HOC*–*COH from two *COHs at adjacent hollow sites,^40^ the high diffusion barrier of *COH (∼0.85 eV)^24^ precludes the emergence of this species under ambient conditions until the surface is covered with *COH. At more negative potentials (vide infra), with the surface coverage of *COH high enough that diffusion is not required prior to coupling, C–C coupling via *COH can proceed. Beyond these non-electroactive pathways, given the long history of invoking *CO as a crucial intermediate, electroactive pathways need to be investigated within this higher-level theory to determine if any direct role is feasible for *CO in C–C coupling on Cu(100). Therefore, in this work, we use ECASPT2 to explore electroactive coupling of two *COs to [OC*–*CO]^(1+δ)–^ and its protonated form [OC*–*COH] ^δ−^, and analyze our findings in terms of their implications for methane versus ethylene production.

## Results

### Coupling Reaction Path

We begin by computing the minimum energy path (MEP) for the electroactive coupling of two *COs. We model the Cu(100) surface with a six-layer 5 × 5 supercell Cu(100) periodic slab and introduce two *COs and an Eigen cation [(H_3_O)­(H_2_O)_3_]^+^ on each side of the slab, where each ion is charge-balanced by an electron added to the metal slab (Figure S1). We also introduce two water molecules on each side of the slab that are directly adsorbed on the surface and form hydrogen bonds with the anionic coupling product (atop *COs do not hydrogen-bond with water). Finally, to replicate the aqueous solvent environment, we used an implicit solvation model^41,42^ with the dielectric constant of pure water (ε = 78.4). We mapped out the MEP within periodic DFT using the PBE XC functional^43^ and semiempirical D3 corrections with Becke-Johnson damping (D3BJ) for van der Waals interactions^44^ (see Methods), which we refer to hereafter as DFT-PBE + D3BJ or in most cases simply as DFT. Figure 1A shows the DFT-predicted stationary (reactant, intermediate, and products) and transition-state structures along the path. The reactant structure has the two *COs at atop sites with C–C distance (*d*_C–C_ = 3.46 Å) and with the H atoms of the two adsorbed water molecules pointing away from the *CO molecules. Before the atop *COs approach each other, both molecules first drift toward the bridge sites, which is a local minimum predicted by DFT (*d*_C–C_ = 3.11 Å). At this structure, one of the H_2_Os of the Eigen cation forms a hydrogen bond with a *CO (this *CO will be protonated at the final product). As the C–C bond forms, the axes of two molecules tilt away from each other (to reduce O–O repulsion) and a transition state emerges with a *d*_C–C_ = 2.16 Å that is still too long to be a bond (typically single C–C bond lengths are ∼ 1.5–1.55 Å; see Table S1 for C–C and C–O bond lengths of reference C_2_ molecules). The C–O bonds also concurrently lengthen to *d*_C–O_ = 1.22–1.23 Å (relative to *CO’s 1.17 Å). At this structure, two of the H_2_Os of the Eigen cation and both adsorbed H_2_Os now form hydrogen bonds with the dimer, which help stabilize the accumulating negative charge. The C–C bond shortens further until *d*_C–C_ reaches 1.46 Å and *d*_C–O_s further increase to 1.30–1.31 Å. The C–C is in between a single and a double bond whereas the C–O bonds are in between a double and a single bond (Table S1). These bond lengths can be explained by three contributing resonance structures (Figure 1B, blue panel). While it is a rather simplistic view of the atomic and electronic structure, it also provides a clue as to the charge state of the dimer. We tentatively assign a formal charge of (1 + δ)– to the structure because in two of the three resonance structures, only one of the Os has a −1 charge, while in one of the three both Os have −1 charge. We thus take this as a signature of electrochemically activated coupling, i.e., the coupling involves an electron transfer (ET). We view the δ charge as a local charge transfer from the four Cu atoms (Cu_4_) at the adsorption site to the OC*–*CO. The idealized formal charges do not necessarily reflect the amount of electrochemical charge transferred; the latter depends also on the charge partitioning scheme used for evaluation. Later, we attempt to quantify both the actual electrochemical charge transfer, which we differentiate from the local charge transfer (δ). Previously, we showed that the neutral OC*–*CO prefers an atop/atop adsorption configuration^40^ whereas here we could only stabilize [OC*–*CO]^(1+δ)–^ at the bridge/bridge configuration, similar to what we find for [HOC*–*CO]^δ−^ (vide infra). This is not surprising because [OC*–*CO]^(1+δ)–^ is closer to [HOC*–*CO]^δ−^ than the neutral OC*–*CO with respect to both atomic and electronic structures, where the latter, because of the C–Cu bonds, is glyoxal-like (OHC–CHO) but very unstable relative to decoupling.^40^ Because [OC*–*CO]^(1+δ)–^ is charged, it is stabilized via a solvent-shared hydronium ion (Figure 1A). Montoya et al. found a similar bridge/bridge structure on both Cu(100) and Cu(111), using the same DFT approximation.^10^ The authors further showed (albeit only for Cu(111)) that the identity of the charge-compensating cation (H^+^, Li^+^, or Na^+^) has little effect on the coupling energetics,^10^ illustrating that such a species may form at any pH. Protonation of [OC*–*CO] ^(1+δ)–^ occurs through a contact ion pair H_2_OH^+^···[OC*–*CO]^(1+δ)–^ as a transition state (Figure 1A). At the point of contact of the two ions, a proton transfers, yielding the second product HOC*–*CO. In this structure, the C–C bond remains in between a single and a double bond although shorter than in [OC*–*CO]^(1+δ)–^ (Table S1). The (unprotonated) C–O bond still is in between a double and a single bond. We illustrate the corresponding resonance structures in Figure 1B, purple panel. We tentatively assign a formal charge of δ− to HOC*–*CO because one of the two resonance structures has a −1 charge at the carbonyl O.

**Figure 1:**
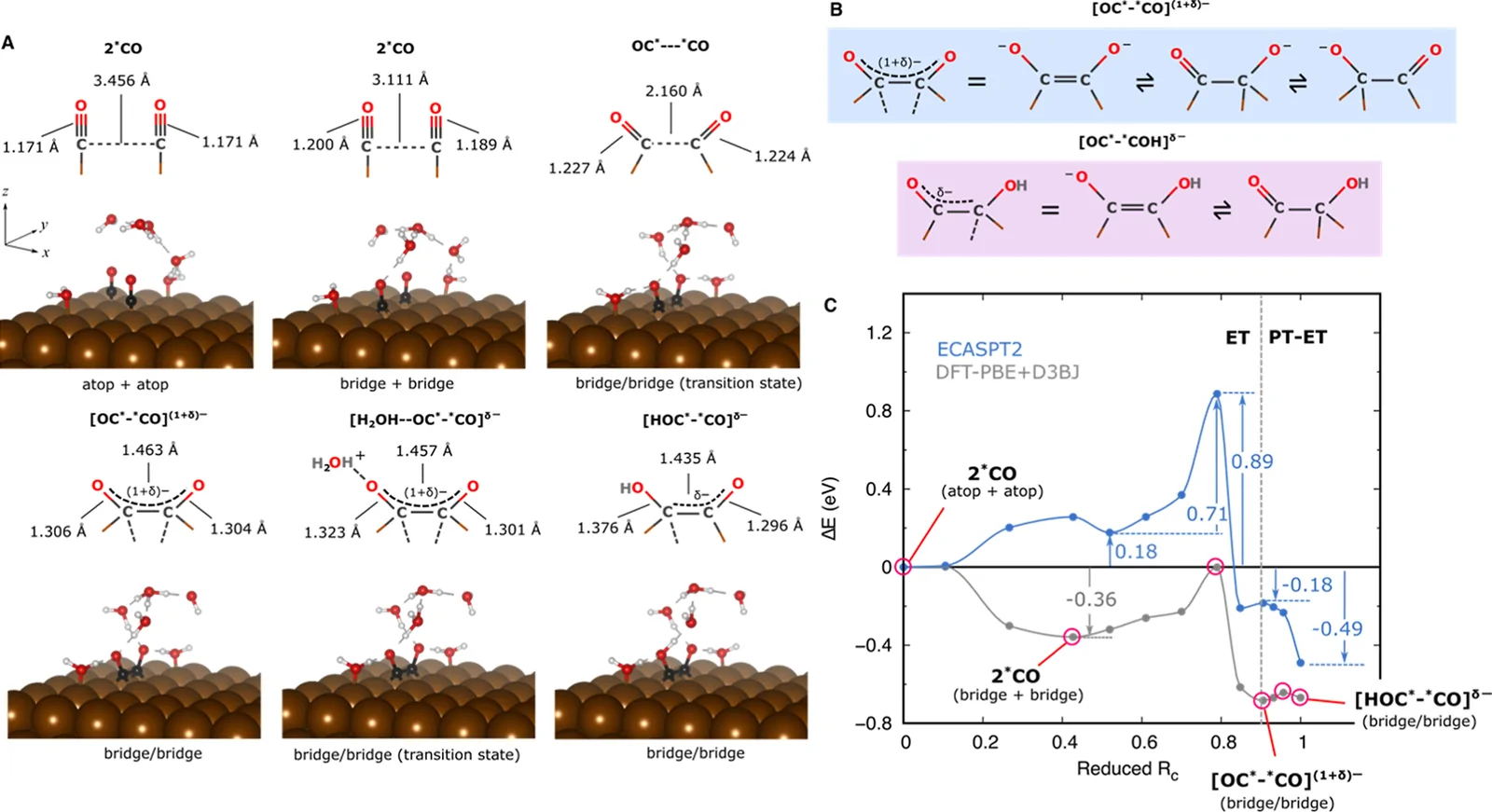
(A) DFT-optimized structures along the minimum-energy coupling path showing atop + atop 2*CO (reactant), bridge + bridge 2*CO (a DFT minimum), transition states (TSs), and products: bridge/bridge [OC*–*CO]^(1+δ)–^ (first TS and product) and bridge/bridge [HOC*–*CO]^δ−^ (second TS and product). The structures are labeled according to the adsorption site of the *COs (or *COH and *CO). If the species are uncoupled or coupled, the label shows “+” or “/”, respectively. The C–C and C–O distances are annotated in their corresponding Lewis structures (determined by consulting bond lengths of reference C_2_ molecules, Table S1). Cu—dark brown spheres, C—black, O—red, and H—white. Vectors: *x* = [011], *y* = [01̅1], *z* = [100]. (B) Electron resonance structures that explain the optimized C–C and C–O bond lengths and illustrate the non-integer formal negative charges on [OC*–*CO]^(1+δ)–^ and [HOC*–*CO]^δ−^. (C) C–C coupling energy curves calculated using DFT-PBE+D3BJ (gray) and ECASPT2 (light blue) in continuum water as a function of a reduced reaction coordinate (*R*_C_), defined in the Methods section. The corresponding structures for the data points marked with red circles are shown in (A) with the DFT-predicted stationary structures labeled. The overall reaction barrier and some reaction energies are marked (numbers on the figures are all in eV). The first (pure ET) and second (PT-ET) parts of the reaction are divided by a dashed vertical line.

Figure 1C shows the corresponding potential energy curves (PECs) calculated within DFT (gray) and ECASPT2 (light blue) at a constant number of electrons in the system. We calculated the ECASPT2 energies within the ECW formalism, where we used DFET to calculate the subsystem interaction (embedding) potential (see Methods and Supplementary Methods (SM) Sections S1 and S2 in the Supporting Information (SI) for details).^34–36^ The DFT PEC illustrates the need to break the reaction into three substeps: (1) diffusion of *COs from atop to bridge, (2) coupling of *COs to [OC*–*CO]^(1+δ)–^, and (3) protonation of [OC*–*CO]^(1+δ)–^ to [HOC*–*CO]^δ−^. We found (vide infra) that substeps (1) + (2) involve only an electron transfer (ET), whereas substep 3 is an ET coupled with proton transfer (PT). Substep (1): as expected, the bridge *COs are lower in energy than the atops within DFT and thus yield a downhill migration to the bridge sites. Substep (2): the coupling of the bridge *COs involves a 0.36 eV DFT barrier. Substep (3): protonation of the coupling product has a small DFT barrier (0.04 eV) nearly equal to this substep’s reaction energy (0.02 eV). Within ECASPT2, the bridge *COs are now higher in energy than the atops (the image to the right of the DFT bridge minimum is an ECASPT2 local minimum that is 0.18 eV higher than the atops). Within ECASPT2, there is now a small barrier for substep (1), ∼0.3 eV, and a much higher barrier for substep (2), 0.71 eV. At substep (3), the DFT minimum is energetically degenerate to the structures found to both of its sides in the path within ECASPT2, rendering this substep barrier free, and with the product much lower in energy, it is also now exothermic.

In ref 38 we had performed similar calculations for the hydrogenation of atop site *CO to hollow site *COH via the PCET with an Eigen cation. To be able to make fully consistent comparisons between the energetics of the electrochemical coupling of *COs and *CO to *COH, we re-evaluated the latter using the same (more refined) model and computational parameters used herein. Supplementary Note 1 and Figure S2 discuss and show the results from the (constant-number-of-electron) simulations for the PCET conversion of *CO to *COH. These calculations yielded DFT and ECASPT2 *CO hydrogenation barriers to form *COH of 0.72 and 1.01 eV, respectively. Both levels of theory predict that the hydrogenation reaction is endothermic: 0.61 (DFT) and 1.01 eV (ECASPT2).

We subsequently converted the internal energies (Table S2) into free energies (Table S3) at 298.15 K for the coupling reactions: substeps (1) + (2)

R1 2CO*+Cu4+e−→[OC*−CO*](1+δ)−+Cu4δ+

and the overall reaction (substeps (1) + (2) + (3))

R2 2CO*+Cu4+e−+H+→[HOC*−CO*]δ−+Cu4δ+

and *CO hydrogenation

R3 CO*+e−+H+→C*OH

Cu_4_ in R1 and R2 consists of the four Cu atoms nearest to the coupling products, as discussed above. We then transformed these free energies from constant electrons to constant potential (vide infra).

### Activation and Reaction Free Energies as Functions of the Electrode Potential

The simulations at constant number of electrons serve as basis for the determination of the free energetics at constant work function (Φ) or equivalently electrode potential (*U*). We determined the potential dependence of the free energy of reaction (Δ*G*_rxn_) and activation (*G* ^‡^) by extending the capacitor model by Chan and Nørskov^45^ for implicitly solvated systems^46^ (Methods and SM Sections S3–S5). We express Δ*G*_rxn_ (*a* = reactant, *b* = product) and *G* ^‡^ (*a* = reactant, *b* = transition state) as linear functions of Φ/*e*

1 $$\Delta G_{a\to b}\left(\Phi \right)={\Delta n}_{e}\left[a\to b\right]\left(\frac{\Phi }{e}-\frac{\left(\Phi_{b}+\Phi_{a}\right)}{2e}\right)+\left(G_{b}\left(\Phi_{b}\right)-G_{a}\left(\Phi_{a}\right)\right)$$

or simply

2 $$\Delta G_{a\to b}\left(\Phi \right)={\Delta n}_{e}\left[a\to b\right]\frac{\Phi }{e}+\Delta G_{a\to b}\left(\Phi =0\right)$$

where the slope Δ*n*_e_[*a* → *b*] is the number of electrons transferred to the adsorbate expressed as charge (or −Δ*q*[*a* → *b*], where *q* is the net charge) when going from structure *a* to *b*. Φ_*a*_ and Φ_*b*_ are the work functions of the slab + adsorbates in solvent in states *a* and *b*. *G*_*b*_(Φ_*b*_) – *G*_*a*_(Φ_*a*_) is simply the change in the free energy from structure *a* to *b* at constant charge. $\Delta G_{a\to b}\left(\Phi =0\right)$ , the vertical axis intercept of eq 2, is thus $\left(G_{b}\left(\Phi_{b}\right)-G_{a}\left(\Phi_{a}\right)\right)-{\Delta n}_{e}\left[a\to b\right]\frac{\left(\Phi_{b}+\Phi_{a}\right)}{2e}$ . Equation 1 stems from the idea that the electrode–electrolyte interface is a capacitor, with the two plates being the electrolyte and the electrode, where one plate has charge +*q* and the other −*q*. Thus, the interface has a capacitance *C* ∼ $-\frac{\Delta q}{\Delta \left(\Phi /e\right)}=\frac{{\Delta n}_{e}}{\Delta \left(\Phi /e\right)}$ per unit area (SM Section S3).^45^ One can infer from eq 1 that at $\Phi =\frac{\left(\Phi_{b}+\Phi_{a}\right)}{2}$ , $\Delta G_{a\to b}\left(\Phi \right)$ is simply the free energy at constant charge, *G*_*b*_(Φ_*b*_) – *G*_*a*_(Φ_*a*_), and that the change in the energy stored into the system relative to $\Phi =\frac{\left(\Phi_{b}+\Phi_{a}\right)}{2}$ is ${\Delta n}_{e}\left[a\to b\right]\left(\frac{\Phi }{e}-\frac{\left(\Phi_{b}+\Phi_{a}\right)}{2e}\right)$ . We used the Bader charge partitioning scheme^47,48^ in our analysis for the values of Δ*n*_e_s (SM Section S4). We determined that the intuitive and physical way to determine Δ*n*_e_ is to consider the 2*COs and Cu_4_ at the adsorption site to be the electroactive unit for the coupling reaction, which becomes ([OC*–*CO]^(1+δ)–^ + Cu_4_^δ+^) and ([HOC*–*CO]^δ−^ + Cu_4_^δ+^) for the first and second products, respectively. By including Cu_4_, we do not include the local bonding charge transfer δ in the electrochemical charge transfer Δ*n*_e_ (SM Section S4, vide infra). For the hydrogenation reaction, on the other hand, the electroactive unit is simply *CO or *COH (SM Section S4).

Table 1 summarizes Δ*n*_e_ and the constant-Φ/*e* Δ*G*_rxn_ and *G* ^‡^ at Φ = 0 V and at Φ/*e* = *U*_RHE_ (pH = 0), where *U*_RHE_ is the absolute potential of the RHE, based on ECASPT2 free energetics (Table S3) of R1, R2, and R3. The calculated Δ*n*_e_s show that R1 is a partial ET with Δ*n*_e_ = 0.6 *e* at the product and Δ*n*_e_ = 0.3 *e* at the transition state. A full electron transfer only occurs after the full reaction R2 with Δ*n*_e_ = 1.1 *e*. Substep (3) is therefore a partial ET (Δ*n*_e_ = 0.5 *e*) coupled with PT. The hydrogenation reaction R3 involves full electron transfer at the transition state (Δ*n*_e_ = 1.2 *e*), where *COH is already fully formed (Supplementary Note 1 and Figure S2).

**Table 1:** Number of Electrons Transferred Δ*n*_e_ and ECASPT2 Reaction Free Energetics at 298.15 K and Φ/*e* = 0 V and Φ/*e* = *U*_RHE_ (*i*.*e*., *U* = 0 vs. RHE and pH = 0) = 4.281 V^a^

reaction	P–R^b^			T–R^b^		
	Δ*n*_e_ ^c^ [*e*]	Δ*G*_rxn_ [eV]		Δ*n*_e_ ^c^ [*e*]	*G* ^‡^ [eV]	
		Φ/*e* = 0	Φ/*e* = *U*_RHE_		Φ/*e* = 0	Φ/*e* = *U*_RHE_
2*CO + Cu_4_ + e^–^ →[OC*–*CO]^(1+δ)–^ + Cu_4_^δ+^	0.59	–2.32	0.23	0.30	–0.13	1.14
2*CO + Cu_4_ + e^–^ + H^+^ →[HOC*–*CO]^δ−^ + Cu_4_^δ+^	1.07	–4.48	0.10	-	-	-
*CO + e^–^ + H^+^ →*COH	1.21	–4.17	0.91	1.20	–4.01	0.93

a *U*_RHE_(pH = 0) = 4.281 V is the experimental value for the absolute potential of the standard hydrogen electrode from ref 49.

b R = reactant, P = product, and T = transition state.

c Calculated from full system DFT-PBE + D3BJ electron density (Eqs S16 or S22 in SM Section S4).

We calculated a net Bader charge (*q*) of −1.52 *e* for [OC*–*CO]^(1+δ)–^ indicating a δ = 0.52, whereas we found a *q* = +0.54 *e* for Cu_4_^δ+^. The *q*s we calculated for [OC*–*CO]^(1+δ)–^ and Cu_4_^δ+^ provide credence to our formal charge assignments and assumption that a local charge transfer between [OC*–*CO] and Cu_4_ is non-electrochemical in nature. We calculated a *q* = −1.03 *e* for [HOC*–*CO]^δ−^, indicating a singly deprotonated *cis*-ethenediol-like character—correspond to its anionic resonance structure (Figure 1B). We found a *q* = +0.57 *e* for Cu_4_^δ+^, illustrating a more delocalized charge compensation for [HOC*–*CO]^δ−^. The *q* value for [HOC*–*CO]^δ−^ (compare charges of [OC*–*CO] and [HOC*–*CO]) of course is consistent with substep (3) being not simply a PT but a partial ET and PT, because if it is only PT, *q* should have been (−1.52 + 1) = −0.52 *e* for [HOC*–*CO]^δ−^. Note that the magnitude of *q* and Δ*n*_e_ do not correspond to each other because Δ*n*_e_ is referenced to the reactant, which also has a non-zero *q*. Δ*n*_e_ also accounts for the number of electrons added concurrently with the addition of a proton. The relationship Δ*q* = – Δ*n*_e_ + Δ*n*_p_, however holds, where Δ*n*_p_ is the change in the number of protons in the molecule because the proton neutralizes the electron that concurrently transfers with it in a PCET reaction (SM Section S4).

At Φ/*e* = *U*_RHE_, i.e., applied potential *U* = 0 vs RHE, and pH = 0, while Δ*G*_rxn_ for the coupling reaction is much lower by 0.7 to 0.8 eV than the hydrogenation reaction, *G* ^‡^ is 0.21 eV lower in the latter than in the former reaction. And because charge transfer is already complete (Δ*n*_e_ ∼ 1*e*) at the transition state in the latter, the activation free energy of the hydrogenation reaction is also more sensitive with the applied potential (the slope of Δ*G* vs Φ/*e* is Δ*n*_e_). Therefore, the electroactive hydrogenation of *CO to *COH should be ∼×3000 faster (exp­[Δ*G* ^‡^/*k*_B_ *T*] = exp­[0.21/0.026]; *k*_B_ = Boltzmann constant, *T* = temperature) than the coupling reaction at room temperature at Φ/*e* = *U*_RHE_. The rate difference should increase even further with negative potentials.

Figure 2 shows the (Φ/*e*)- and pH-dependence of *G* ^‡^ (top) and Δ*G*_rxn_ (bottom) for the aforementioned coupling and hydrogenation reactions (as labeled). The steps that involve PT exhibit pH-dependence, in which case, we evaluated those free energies at pH = 0, 7, and 13. Because RHE is a pH-dependent scale, we also show the *U* vs RHE scale, i.e., *U* = (Φ/*e*) – *U*_RHE_, at the same three pHs (note that we use the experimental *U*_RHE_ = 4.281 V at pH = 0).^49^

**Figure 2:**
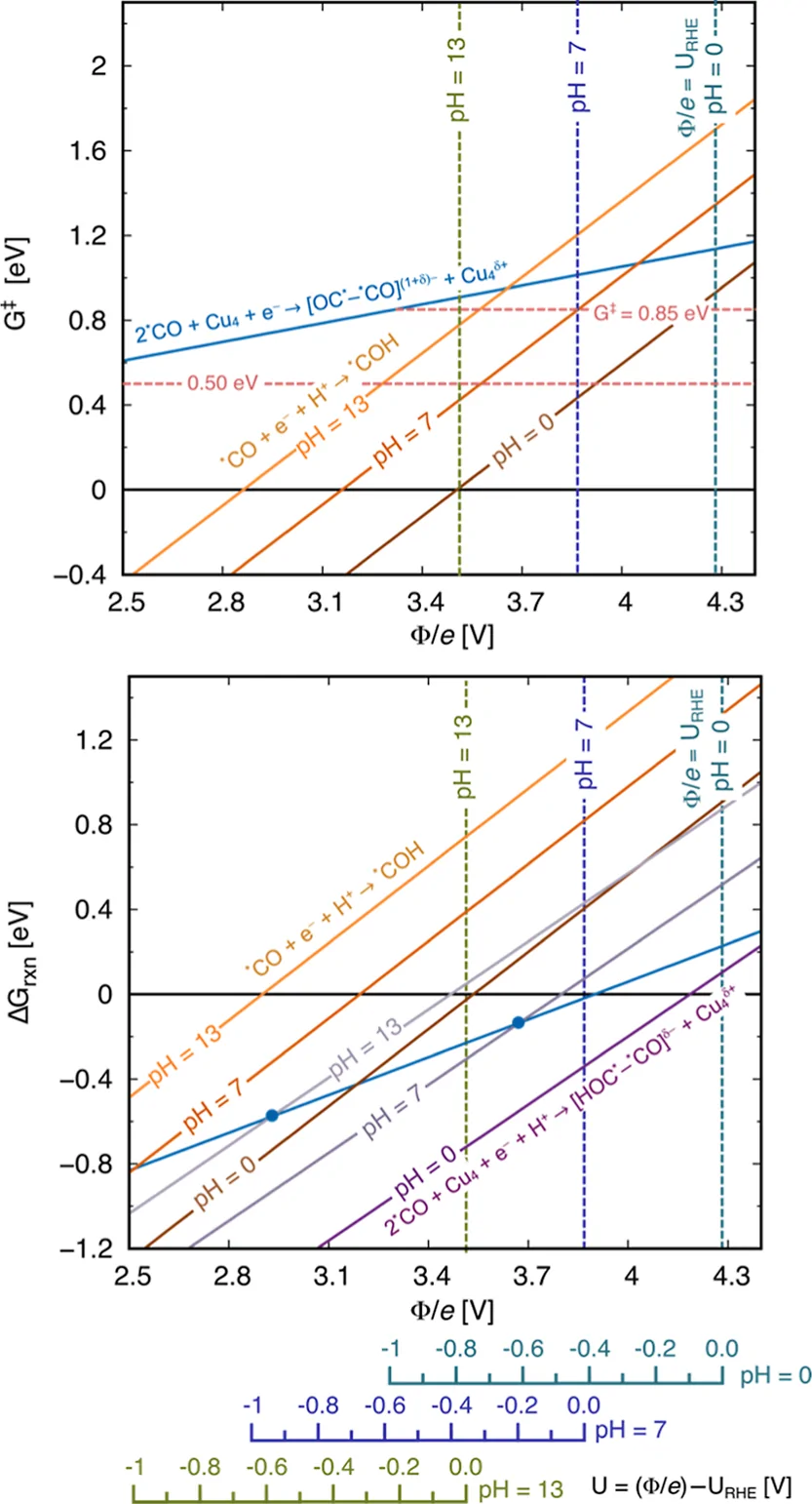
ECASPT2 free energies of activation (*G* ^‡^, top) and reaction (Δ*G*_rxn_, bottom) as functions of surface potential (Φ/*e*) and pH for both the electroactive coupling of *CO and reduction of *CO to *COH. The pH-dependent RHE scales (*U* = Φ/*e* – *U*_RHE_) at pH = 0, 7, and 13 are also shown in the bottom horizontal axis (shifted horizontally from pH = 0 by – [0.0592 × pH]). Top, [OC*–*CO]^(1+δ)–^ and [HOC*–*CO]^δ−^ share a common rate-determining barrier, thus, only the former, which occurs first, is shown in the *G* ^‡^ plot (light blue). The barrier for *CO to *COH exhibits pH dependence (brown to orange) because it is a PT-ET process (shifted vertically from pH = 0 by 0.0592 × pH), whereas the coupling of 2*CO to [OC*–*CO]^(1+δ)–^ does not because it is only an ET step. Bottom, free energies of reaction to form [HOC*–*CO]^δ−^ (purple to gray) and *COH (brown to orange) from atop *CO are shown at different pHs (0.0592 × pH). The pH-independent formation of [OC*–*CO]^(1+δ)–^ (light blue) is shown, and its intersection points with the [HOC*–*CO]^δ−^ line at pHs = 7 and 13 are marked (light blue points). At Φ/*e* greater than (i.e., *U* less negative than) the value at the intersection points, [OC*–*CO]^(1+δ)–^ becomes more thermodynamically stable than [HOC*–*CO]^δ−^ for a given pH. Vertical dashed lines mark *U* = Φ/*e* – *U*_RHE_ = 0 V for different pHs (as labeled). Horizontal dashed lines mark *G* ^‡^ = 0.5 (fast reaction) and 0.85 eV (*COH diffusion barrier).

The *G* ^‡^ plot in Figure 2 shows that the hydrogenation reaction to *COH should occur much faster than the coupling to [OC*–*CO]^(1+δ)–^ at all pHs for *U* < 0 vs RHE. The [OC*–*CO]^(1+δ)–^ line intersects with the *COH lines at *U* ∼ +0.2–0.3 V vs RHE, which indicate that the coupling reaction can be made faster than the hydrogenation reaction at more positive values (a little less positive at higher pHs), but the absolute rate will be low, given the high free energy barrier (>0.8 eV at potentials > RHE). From pH = 0 to 13, *G* ^‡^ for the hydrogenation ranges from 0.9 to 0.85 eV at *U* = 0 and crosses 0.0 eV at *U* ∼ −0.8 to −0.65 V vs RHE (note that G^‡^ cannot be < 0). For the coupling reaction, *G* ^‡^ goes from 1.1 to 0.9 at *U* = 0 vs RHE and from 0.85 to 0.6 eV at *U* = −1 V vs RHE from pH = 0 to 13. Although Δ*n*_e_ is smaller for the coupling than the hydrogenation reaction, *G* ^‡^ of the former is more sensitive to pH for a given *U* because coupling involves only ET (no PT) at the transition state (pH-independent) whereas the reference scale (RHE) is pH-dependent. We mark *G* ^‡^ = 0.5 eV in the plot, the value at which the Eyring rate constant^50^ $k=\frac{k_{B}T}{h}e^{-G^{‡}/k_{B}T}$ (*h* = Planck constant) or turnover frequency becomes 2.7 × 10^4^ molecules s^–1^ (considered fast catalytic turnover). The *COH lines intersect this value at *U* ∼ −0.35 V vs RHE, which indicates (as we already concluded in ref 38) fast *CO conversion to *COH even under moderately reducing potentials.

The Δ*G*_rxn_ plot in Figure 2 shows an opposite trend for the thermodynamic favorability compared to the kinetics. The Δ*G*_rxn_ plot now also shows the formation of [HOC*–*CO]^δ−^ from *COs, which is pH-dependent because it involves PT. Δ*G*_rxn_ of the hydrogenation reaction is now always higher than either or both of the coupling reactions for all pHs and *U* (note that one can only directly compare the free energy lines for the same pH between different ET-PT reactions; the free energy line of an ET reaction can be compared to an ET-PT reaction at any pH). At pH = 0, [HOC*–*CO]^δ−^ is always lower in energy than [OC*–*CO]^(1+δ)–^ at *U* < 0 V vs RHE. At pH = 7 and 13, the deprotonated form is more stable than [HOC*–*CO]^δ−^ at *U* = 0 V vs RHE until *U* = −0.2 and −0.6 V, respectively, showing that (as expected) at higher pH, the magnitude of negative potential needed to offset the thermodynamic penalty to draw a proton from H_2_O is also higher. Δ*G*_rxn_ is also ≲ 0.1 eV for the more stable coupling product for *U* ≤ 0 vs RHE for all pH. Δ*G*_rxn_ of the hydrogenation reaction crosses zero approximately +0.05 V of *U* where *G* ^‡^ = 0, namely, at ∼−0.75, −0.7, and −0.6 V vs RHE at pH = 0, 7, and 13, respectively. Note that if only thermodynamics were used to assess the catalytic activity of Cu(100), then one would mistakenly conclude that electrochemical *CO coupling is more favorable than hydrogenation to *COH.

## Discussion

A hypothetical molecule resulting from the coupling of two COs in the gas phase is ethylenedione, which has yet to be experimentally synthesized. A report of its (short-lived) realization^51^ was subsequently disproven.^52^ The molecule’s radical anionic form (O=C–C=O)^•–^ has been claimed to be generated via H_2_^+^ abstraction from the relatively stable glyoxal molecule (OHC–CHO) through a collision with an unstable radical anion O^–^.^51^ The salts of the dianion (^−^O–C≡C–O^–^), or acetylenediolate, may be synthesized from the reduction of CO with very strong reducing metals, such as, alkali metals, in the absence of acidic hydrogen.^53,54^ For these highly unstable species to emerge during ECO/CO_2_R on a metal electrode, the metal (in the presence of excess electrons) must form stabilizing bonds with them. In ref 40, we calculated, also within ECASPT2, non-electroactive free energy barriers for the coupling of *CO and *COH to neutral OC*–*CO, [HOC*–*CO]^δ−^, and HOC*–*COH of 1.74, 1.19, and 0.27 eV, respectively. We also found that the neutral glyoxal-like OC*–*CO (with Cu replacing the role of H) is not a local minimum. Contrary to what DFT suggests, ECASPT2 predicts that kinetics inhibits the formation of both OC*–*CO and [HOC*–*CO]^δ−^, although the latter is thermodynamically stable (Δ*G*_rxn_ = −0.2 eV).^40^ The ECASPT2 trend in kinetic unfavorability of the formation of these species is more in keeping with the known stability of their gas-phase counterparts. Furthermore, in the current work we found that allowing the electroactive coupling to occur first to yield [OC*–*CO]^(1+δ)–^ may enable formation of [HOC*–*CO]^δ−^ from *COs at a lower G^‡^ than the direct coupling of *CO and a *COH, in a way that may also be tuned with applied potential (vide supra), e.g., with *G* ^‡^ ranging from 0.84, 0.72, and 0.61 eV to 1.14, 1.01, 0.91 eV at pH = 0, 7, and 13, respectively, when going from *U* = −1 to 0 V vs RHE (Figure 2, top panel). Protonation can then easily follow to yield a highly thermodynamically favorable [HOC*–*CO]^δ−^. However, given that the coupling reaction is kinetically limited and would never reach the thermodynamic limit because of a faster competing hydrogenation reaction to *COHs that consumes *CO (*G* ^‡^ = 0.5–0.9 eV at *U* ∼ −0.35–0 V vs RHE for all pHs and becomes 0 at *U* ∼ −0.7 V vs RHE), then the thermodynamics of coupling becomes irrelevant in the kinetics of ECO/CO_2_R on Cu(100). Note that the G^‡^ of the coupling to [OC*–*CO]^(1+δ)–^ does not become lower than the diffusion barrier for *COH (∼0.85 eV)^38^ until *U* ∼−1, −0.55, and −0.2 V vs RHE at pH = 0, 7, and 13 (Figure 2, top panel), which are already more negative or close to *U* where *G* ^‡^ of hydrogenation crosses 0.5 eV (*U* ∼−0.35 V vs RHE for all three pHs). Thus, even when *COH formation does not completely deplete the *COs, electrochemical coupling of *COs cannot be a significant route for C–C bond formation.

Schouten et al. observed two possible C_2_ pathways on Cu(100) based on the appearance of ethylene product.^5–7^ The first appears at *U* ∼−0.4 V vs RHE at pH = 7, peaking at ∼−0.6 V before dying off, with the second emerging at −0.8 V at the same pH. At the second surge, methane was observed to competitively form. At pH = 13, the first surge shifts 0.1 V to *U* ∼−0.3 V with a peak at ∼−0.45 V before dying off, with no second peak at more negative potentials. The formation of HOC*–*COH could partly explain the second surge at pH = 7 and −0.8 V vs RHE (*G* ^‡^ coupling of 0.27 eV, vide supra)^40^ because Δ*G*_rxn_ for the formation of *COH becomes ≲ 0 eV at *U* ∼ −0.75 to –0.6 V vs RHE and lower (more negative) from pH = 0 to 13, and thus *COH may accumulate and enable coupling without the need for its diffusion. The formation of HOC*–*COH naturally competes with the hydrogenation of *COH that eventually yields probable precursors to methane. Coupling of *COHs to HOC*–*COH does not mean other C–C coupling pathways involving more hydrogenated C_1_ species will not also contribute to ethylene production—one would expect more channels for coupling to become available at more negative potentials as more partially hydrogenated C_1_ species form. Additional (embedded) correlated wavefunction studies on hydrogenated species beyond *COH (*CH_*x*_OH or *CH_*y*_) and their coupling would help establish a fuller picture of ethylene and methane production on pristine Cu(100), especially at moderate potentials corresponding to the first surge,^5–7^ i.e., less negative than *U* ∼ −0.75 to –0.6 V vs RHE. The observed pH-dependence of the onset potential of the first ethylene surge against a pH-dependent reference scale (e.g., RHE), indicates that coupling at low to moderately reducing potentials likely only involves as a rate-determining step (RDS) an ET step, i.e., the RDS does not involve a PT. It is conceivable that at least one of the *CH_*x*_OH and *CH_*y*_ species, which ought to have a higher surface mobility than *COH (kinetically favored to form at *U* ∼ −0.4 V and lower (more negative) but diffusion-limited by its 0.85 eV hopping barrier), given their lower coordination with the surface Cu atoms, could facilitate electroactive C–C bond formation without the need for a concurrent PT.

Finally, we have ignored the role of *CHO on Cu(100) due to our prior work indicating its formation from *CO is not competitive against *COH on this surface,^38^ although many other (DFT-based) theoretical studies have argued that coupling of *CHO to OHC*–*CO or adsorbed glyoxal OHC*–*CHO is also a feasible pathway for ethene production.^8,9,11,12,14,16,55^ A recent experiment by Reichert et al. has shown that using glyoxal instead of CO or CO_2_ as reactant for electroreduction does not lead to any meaningful amounts of ethylene, discounting the role of *CHO coupling in C_2_ production via Cu ECO/CO_2_R.^56^ This adds credence to our prior conclusion that *CHO is not competitively produced from *CO on Cu(100) and therefore not relevant in the observed C_2_ production, with Reichert et al.’s work showing that if OHC*–*CHO does form, ECO/CO_2_R would not yield ethylene as one of the primary hydrocarbon products.^38^

## Summary and Conclusions

In Figure 3A, we plot the G^‡^ s of all the coupling reactions we studied so far on Cu(100) within ECASPT2. We took the non-electroactive *G* ^‡^s (i.e., those that do not change with Φ/*e*) from ref 40. Combining the data presented in Figures 2 and 3A, we present in Figure 3B a summary of the pathways and *G* ^‡^s linking the species to ethylene or methane. The figure shows the non-electroactive and electroactive coupling of *CO and *COH, and the electroactive hydrogenation of *CO to *COH and *CHO. Steps that are slow (*G* ^‡^ > 0.8 eV), moderate (0.5 < *G* ^‡^ < 0.8 eV), and fast (*G* ^‡^ < 0.5 eV) are color coded by red, orange, and green arrows, respectively.

**Figure 3:**
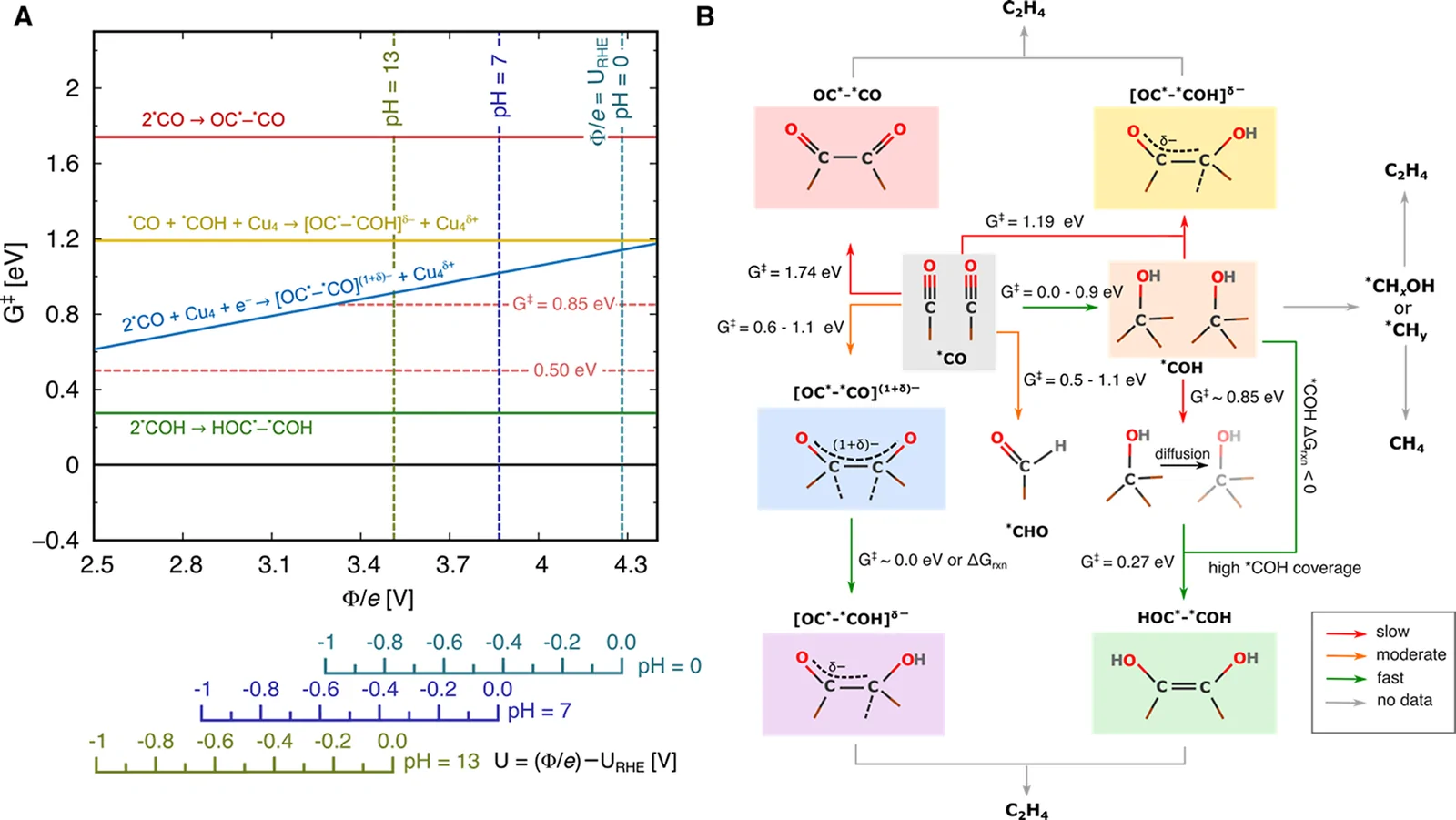
(A) ECASPT2 *G* ^‡^ vs Φ/*e* of the coupling reactions only (as labeled). The *G* ^‡^ line (light blue) for [OC*–*CO]^(1+δ)–^ is taken from Figure 2, top panel. Horizontal dashed lines mark *G* ^‡^ = 0.5 (fast reaction) and 0.85 eV (*COH diffusion barrier). (B) Overview of ECO/CO_2_R pathway originating from *CO on Cu(100) (gray box) from ECASPT2 with ethylene and methane as products. *G* ^‡^ of the elementary steps are shown with arrows, color-coded based on their qualitative rates: slow (*G* ^‡^ > 0.8 eV) in red, moderate (0.5 < *G* ^‡^ < 0.8 eV) in orange, and fast (*G* ^‡^ < 0.5 eV) in green. In both panels, *G* ^‡^ to form the species were either calculated here or taken from refs 38,40 . In (B), the colors of the boxes correspond to line colors used to represent the species in panel (A) and Figure 2. A range of *G* ^‡^ values are given for the electroactive steps in (B), with the lower and upper bound corresponding to *U* = 0 V at pH = 0 and *U* = −1 V at pH = 13 vs RHE.

Hydrogenation of *CO to *COH is the fastest route of consumption of *CO, with the coupling to OC*–*CO and [OC*–*CO]^(1+δ)–^ throttled by higher barriers, also shutting off [HOC*–*CO]^δ−^ via *CO. *CHO also cannot form competitively. Before *G* ^‡^ crosses 0.5 eV for *COH (U ≳ −0.35 V (less negative) vs RHE), *CO and *COH may coexist, but even then direct coupling to [HOC*–*CO]^δ−^ is impeded by a high barrier. Diffusion of *COH is unlikely at ambient temperatures and thus the instantaneous coupling to HOC*–*COH is only possible at high *COH coverages (at potentials more negative than the potentials at which Δ*G*_rxn_ for hydrogenation crosses 0 eV, i.e., ∼−0.7 V vs RHE). HOC*–*COH thus can be a precursor for ethene at very reducing potentials, whereas a more hydrogenated, more mobile form of *COH, i.e., *CH_*x*_OH or *CH_*y*_, must be involved in C–C bond formation at less reducing potentials. Our results also suggest that morphologically tailoring Cu(100), e.g., via defect engineering, to enable fast and thermodynamically favorable buildup of *COH and facile diffusion and dimerization subsequently, could be a route to practical electrochemical ethylene production from CO_2_ on Cu even at low overpotentials.

Based on our prior bodies of work^38,40^ on *CO hydrogenation via PCET and non-electroactive coupling involving *CO, and now electroactive *CO coupling pathways, it is clear that coupling involving *CO could never be as competitive as hydrogenation to *COH, and thus *CO unlikely participates in C–C formation on pristine Cu(100) at low and especially not at highly reducing potentials. Rather, at the more negative potentials, the competitive production of ethylene and methane is due to *COH coupling and *COH hydrogenation, respectively. To further explain the experimentally observed preferential conversion of CO/CO_2_ to methane and ethylene, and competition between these products, our study suggests that we should pivot our focus to the more hydrogenated species, especially at moderate potentials, where the earlier hypothesis of CO–CO coupling must be discarded for a new mechanism, perhaps one involving *COH and a more highly hydrogenated species.

In closing, our work suggests that what sets apart Cu from other metals in producing hydrocarbons from CO_2_ is not its ability to bind *CO favorably to enable their coupling. Indeed many metals can stably bind *CO as well (Ag) or even more strongly than Cu (Co, Ni, Rh, Pd, and Pt),^57–63^ and yet they produce *CO as primary terminal product.^1^ It is more likely that the efficient (multiple) hydrogenation(s) of CO unlocks Cu’s unique ability to produce C_2+_ hydrocarbons.

## Methods

### Periodic DFT Simulations

We used the Vienna Ab-Initio Simulation Package (VASP)^64,65^ version 6.4.1 compiled with the VTST,^66^ VASP-sol,^41,42^ and DFET^67^ subroutines to perform periodic spin-restricted Kohn–Sham DFT calculations with the PBE XC functional.^43^ Together with the projector augmented-wave (PAW) method,^68^ we used the standard PAW potentials to represent the nuclei and frozen core electrons of H, C, O, and Cu, and thus only self-consistently optimized their 1s, (2s,2p), (2s,2p), and (4s,3d) valence electrons, respectively. Accordingly, we used a planewave basis set with a kinetic energy cutoff of 660 eV in representing the aforementioned valence electrons. We used finite *k*-point sampling of a 4 × 4 × 1 Monkhorst–Pack *k*-point mesh^69^ to sample the Brillouin zone for the (5 × 5) metal slab supercell, resulting in an in-plane reciprocal lattice spacing of ∼0.02 2πÅ^–1^. To aid electronic convergence, we introduced electronic smearing using the Methfessel–Paxton scheme^70^ with a width of 0.09 eV.

To simulate the electroactive reactions, we introduced a molecular cluster ((H_3_O)­(H_2_O)_3_) above the adsorbates that self-ionizes into a positive hydronium ion, transferring an electron to the metal slab. We performed such simulations in the presence of continuum water to stabilize the ion, where we used one-sided (1S) and two-sided (2S) slab models. The 1S (100 Cu atoms) and 2S (150 Cu atoms) models are four- and six-layer (5 × 5) supercell slabs, respectively. Figure S1 shows the 2S model. We took the optimized lattice constant of face-centered cubic Cu (conventional cubic cell constant *a* = 3.567 Å) from ref 71. The Cu(100) slab thus has in-plane lattice vectors of 12.611 Å × 12.611 Å. The surface normal vectors have lengths of 25 and 34 Å for the 1S and 2S models, respectively. These surface normal vectors correspond to 19.7 and 25.1 Å between the outer layers of the slabs through the vacuum of the 1S and 2S models, respectively. All three lattice vectors are orthogonal to each other. We used the same 1S model in ref 40 to study non-electroactive coupling of *CO and *COH on Cu(100).

We optimized the atoms in the slabs while fixing the lattice constants. We also fixed the bottom two and middle two Cu layers to their bulk structure for 1S and 2S slabs, respectively. We relaxed all stationary structures until the maximum absolute atomic force of 0.03 eV/Å is reached for all the atoms allowed to move. We used the climbing image nudged elastic band (CI NEB) method^72^ to determine the reaction path and thus the transition states. We used a spring force constant of 5 eV/Å^2^. We set a force threshold of 0.05 eV/Å for the convergence of the NEB images to balance accuracy and cost. We performed simulations using the 1S model as an efficient method to generate initial guess structures for the 2S model and thus improve the structure convergence rate of the latter model, which is a more costly simulation. We define the reaction coordinate (*R*_C_) along the MEP as

$$R_{C}\left(n\right)=\sum_{j=1}^{n}\sqrt{\sum_{i=1}^{N}{\left(x_{i,j}-x_{i,j-1}\right)}^{2}+{\left(y_{i,j}-y_{i,j-1}\right)}^{2}+{\left(z_{i,j}-z_{i,j-1}\right)}^{2}}$$

where *n* is the image index in CI-NEB (*n* = 0 for reactant), *N* is the total number of atoms allowed to move, and (*x*_*i*,*j*_,*y*_*i*,*j*_,*z*_*i*,*j*_) are the Cartesian coordinates of atom *i* in the *j*th CI-NEB image. We calculated reduced *R*_C_ as *R*_C_(*n*)/*R*_C_(*n* = product), which is unity at the product.

In the 2S model, which is necessary to avoid unphysical slab polarization that occurs for asymmetric metal slabs with excess charge in the presence of a polarizable medium, we made the two surfaces to be identical (related through mirror plus translation symmetry operations) in the initialization. To ensure the two surfaces evolve equivalently, we symmetrize the two surfaces (through mirror plus translation symmetric replication of the top side of the slab at the bottom side) whenever ionic relaxation or CI-NEB routine terminates, i.e., either the calculation runs out of time, or the maximum number of ionic steps is met. Finally, when force convergence is met during either ionic optimization or CI-NEB routine, we performed a final symmetrization of the two surfaces followed by the final set of structural optimization steps, at which point only a few ionic steps are required to re-establish force convergence and effectively guarantee that the two surface are the same or almost the same.

Figure S3A shows the structures obtained from the 1S model for the coupling reaction, which are similar to the final structures obtained from the 2S model (Figure 1A). The energetic differences between the two model sizes are, however, not trivial (Figure S3B). We also tested the effect of simply using vacuum in the simulation and found the vacuum energetics to be unphysical (Figure S3C). Additionally, we tested adding screening effects of dissolved ions as part of the implicit solvent in the coupling reaction. We did so by introducing an implicit electrolyte model with a Debye screening length (λ_D_) of 3.04 Å (λ_D_ is effectively set to ∞ for pure water in the model), which corresponds to a 1 M monovalent ionic solution^42^ (Figure S3C). As expected, because the electrons in the 2S metal slab are already effectively screened by explicit hydronium ions above the surfaces, the effect of additional screening in the implicit solvent is negligible (<0.002 eV). Furthermore, water’s dielectric constant is known to drop near electrodes; e.g., a very recent experiment showed that the dielectric constant of water can be as low as ∼35–40 due to interfacial confinement of water.^73^ We therefore reevaluated the reaction energy curve at ε­(H_2_O) = 40 (Figure S3C). A 50% reduction in ε­(H_2_O) had <0.1 eV effect on the barrier and reaction energy, smaller than the energy differences we analyze in this work (e.g., Figure 2 shows that the difference in *G* ^‡^s between the coupling and hydrogenation reactions is >0.2 eV).

We refer the readers to ref 40 for the method we used to include finite temperature corrections in order to convert the reaction and activation internal energies to reaction and activation Gibbs free energies.

### ECASPT2 Energy Correction

We use the pristine 100-Cu-atom 1S model, which we partitioned into a Cu_17_ cluster as the reactive site, surrounded by a periodic Cu_83_ environment. We chose the cluster size and shape by ensuring that all direct interactions of the adsorbates with the surface (reactant, product, and all structures along the reaction path) are contained within the cluster, and the cluster with adsorbates contain an even number of electrons (the full system is a spin singlet). Previously we showed that as long as this is the case, then the energies are insensitive to cluster size.^74^ We derived the interaction of the cluster and environment as an optimized effective potential, i.e., an embedding potential (*V*_emb_), within the DFET formalism using the periodic DFT-code VASP (SM Section S1).^36,67,75^ We then performed the nonperiodic embedded cluster calculations using Molpro v. 2024,^76^ wherein we used atom-centered Gaussian-type basis sets, namely, the all-electron correlation-consistent polarizable valence double-ζ (cc-pvdz)^77^ for H, C, and O; and the LANL2DZ^78^ basis set and accompanying 10-electron–core effective core potential (ECP) for Cu.^79^ We transplanted the DFT-optimized adsorbate structures from the top side of the 2S model onto the Cu_17_ cluster. We added *V*_emb_ into the one-electron Hamiltonian by first transforming *V*_emb_ from values on a Cartesian grid to projections on a Gaussian basis using our embedding integral generator code.^80^

We express the full system solvated ECASPT2 energy (*E*_ECASPT2_^full^) as

$$E_{ECASPT2}^{full}=\frac{E_{DFT‐PBE+D3BJ}^{full}\left(solv\right)-E_{D3BJ}^{full}}{2}+\left(E_{eCASPT2}^{cl}\left[V_{emb}\left(r\right),vac\right]-E_{eDFT‐PBE}^{cl}\left[V_{emb}\left(r\right),vac\right]\right)$$

where the second term in parentheses serves as a regional correction to the first term. *E*_eCASPT2_^cl^[*V*_emb_,*vac*] and *E*_eDFT‑PBE_^cl^[*V*_emb_,*vac*] are respectively the (nonperiodic) embedded cluster (*cl*) energies within CASPT2 and DFT-PBE in vacuum calculated using Molpro. *E*_DFT‑PBE+D3BJ_^full^(solv) and *E*_D3BJ_^full^ are the total energy and only the D3BJ van der Waals energy, respectively, of the solvated full periodic 2S slab with the adsorbates calculated from VASP. We subtract the latter from the former because the correction term should contain the correct van der Waals energy from CASPT2 that is not captured within DFT-PBE. The factor 1/2 appears in the first term because we used a 2S slab model. We performed the embedded cluster calculations in vacuum because use of any solvation model would lead to unphysical solvation of the metal cluster that would otherwise be buried within the slab (solvation effects are accounted for in the full slab models).

We generated the multiconfigurational reference wavefunctions from the embedded complete active space self-consistent field (ECASSCF) method^81,82^ using an active space (AS) of 14 electrons in 14 orbitals or (14e,14o). Figures S4 and S5 show the ECASSCF AS natural orbitals for the coupling and hydrogenation reactions, respectively. We followed ECASSCF with PT2 corrections that include excitations to or from orbitals not included in the CASSCF AS except for the core 1*s* of C and O, and outer core 3*s* and 3*p* not included in the ECP of Cu. SM Section S2 provides further information about the ECASSCF and ECASPT2 calculations. We estimated the level of convergence of the ECASPT2 energetics for the electrochemical coupling of COs with respect to AS size by also evaluating the ECASPT2 energies with an AS of (10e,10o). Removing two pairs of occupied and empty orbitals (instead of only one) from AS­(14e,14o) retains equivalent electronic descriptions of the two *COs. The barrier (increased) and reaction energies (lowered) differ by ∼0.1 eV between AS­(10e,10o) and AS­(14e,14o)—Figure S6. We therefore expect convergence errors for the energetics that are ∼0.1 eV or better for our AS (14e,14o) simulations when compared to larger ASs. This is because the orbitals added to yield (14e,14o) from (10e,10o) have occupation numbers ∼1.98 *e* and ∼0.02 *e* for the occupied and virtual orbitals, respectively. Small deviations from occupation numbers of 2 and 0 for the larger AS indicate that static correlation will become even less important for any adsorbate orbitals added beyond (14e,14o).

Overall, we estimate errors in the ECASPT2 internal energies originating from model size,^74^ simulation parameters (e.g., active space—vide supra), and solvation model (vide supra) to be around a tenth of an eV. The differences in *G* ^‡^s between the coupling and hydrogenation reactions are ∼0.2 eV at *U* = 0 vs RHE at any pH, which increases further with *U* < 0 vs RHE due to the greater sensitivity of CO hydrogenation to applied potential (Figure 2). Furthermore, the difference between DFT-PBE+D3BJ and ECASPT2 in the calculated barrier for the electrochemical coupling reaction (Figure 1C) is far greater than this error estimate.

### Potential Dependence of the Free Energies

We use the capacitor model by Chan and Nørskov^45^ applied to implicitly solvated systems to transform the free energetics from constant charge to constant Φ (SM Sections S3). To do the transformation, we determined first the work function Φ of the slabs with adsorbates relative to the implicit solvent potential (Φ – Φ_solv_) by taking the difference between the Fermi level of the former and the electrostatic potential of the latter. To do so, we calculated the average total electrostatic potential, i.e., Hartree (*V*_H_) plus ionic (*V*_ion_), as a function of the surface normal vector coordinate using a high precision real-space fast Fourier transform (FFT) grid (PREC = accurate in VASP) for the relevant stationary and transition state structures of both the coupling (Figure S7 and Table S4) and hydrogenation reactions (Figure S8 and Table S4). Using the electron density evaluated at the same high precision FFT grid, we employed the Bader real-space charge partitioning method^47,48^ and quantified Δ*n*_e_ by considering different atom partitioning schemes including the new scheme used here (SM Section S4 and Table S5). Finally, to determine Φ_solv_ and thus calculate the absolute rather than relative Φs, we first used the computational hydrogen electrode (CHE) for both reactions R2 and R3 to find (Φ – Φ_solv_) for which Φ – Φ_solv_ = *eU*_RHE_ – Φ_solv_.^37,38,46^ Using the empirical *U*_RHE_ = 4.281 V,^49^ we calculated Φ_solv_ (SM Section S5 and Table S6). We then used Φ_solv_ to calculate the Φs (Table S7).

## Supplementary Material



## Notes

Supplementary methods; supplementary note; images of the 2S slab model; *CO hydrogenation to *COH pathway and PEC; comparison between the calculated energetics using the 1S vs 2S models, with vs without implicit electrolyte, and bulk vs interfacial water dielectric constants; ECASSCF active space natural orbitals and occupation numbers; sensitivity of PEC with AS size for the electrochemical coupling reaction; electrostatic potential plots; gas-phase C_2_ structural references; reaction and activation internal and free energies at constant number of electrons from DFT and ECASPT2; work functions vs implicit solvent and amount of charge transfer for the hydrogenation and coupling reactions; internal and free energies of reaction for *COH and [OC*–*CO]^(1+δ)–^ formation within the CHE formalism; work functions vs vacuum for the hydrogenation and coupling reactions (PDF)
