# Supplementary material for: C–C Bond Formation during Electrochemical CO2 Reduction on Pristine Cu(100) Unlikely to Involve Adsorbed CO at Any Potential
Source: J Am Chem Soc. 2026 Feb 12;148(7):7415–25. doi: 10.1021/jacs.5c20300 (PMC13730435; doi:10.1021/jacs.5c20300)
Supplement: Supplementary file 1 [file ja5c20300_si_001.pdf]

Supplementary Information

C-C bond formation during electrochemical CO_2 reduction on pristine Cu(100) unlikely to involve adsorbed CO at any potential

John Mark P. Martirez[1,*] and Emily A. Carter[1,2,*]

[1]Applied Materials and Sustainability Sciences, Princeton Plasma Physics Laboratory, Princeton, New Jersey, 08543-0451, United States

[2]Department of Mechanical and Aerospace Engineering and the Andlinger Center for Energy and the Environment, Princeton University, Princeton, New Jersey 08544-5263, United States

*Corresponding authors: martirez@pppl.gov, eac@princeton.edu

Supplementary Methods

S1. V_{emb} optimization within DFET. To derive the interaction of the clusters (representing active surface sites and their subsurface neighbors) with their respective (extended crystalline) environment, we employed density functional embedding theory (DFET)[1] where we solve for an optimized effective potential (OEP) by using a two-component extension of the Wu-Yang functional.[2] The unique OEP that mutually acts on the subsystems serve to embed them and thus we refer to it as the embedding potential or V_{emb}. To calculate V_{emb} we maximize the scalar quantity W or equivalently minimize $-W$ for a full system partitioned into two subsystems: cluster and environment

$$-W = \left\{\int V_{emb}\, \rho^{full}(r)dr\right\} - E^{cl}[\rho^{cl}(r), V_{emb}] - E^{env}[\rho^{env}(r), V_{emb}], \qquad \textbf{Eqn. S1}$$

where E and $\rho(r)$ are the total energy and electron density, respectively. The superscripts indicate from which system the quantity is calculated from, namely, *cl* = cluster, *env* = environment, or *full* = full system. Both $E^{cl}$and E^{env} are functionals of $\rho(r)$ and V_{emb}. V_{emb} influences the energy and $\rho(r)$ of the embedded fragments via their respective Hamiltonians ($H°_{elec}$): $H^{emb}_{elec} = H°_{elec} + V_{emb}$. The functional derivative of **Eqn. S1** with respect to V_{emb} yields

$$-\frac{\delta W}{\delta V_{emb}(r)} = \frac{\delta E^{full}_{DFT}}{\delta V_{emb}(r)} - \left(\frac{\delta E^{cl}_{eDFT}}{\delta V_{emb}(r)} + \frac{\delta E^{env}_{eDFT}}{\delta V_{emb}(r)}\right) \qquad \textbf{Eqn. S2}$$

or

$$-\frac{\delta W}{\delta V_{emb}(r)} = \rho^{full}(r) - \rho^{cl}(r) - \rho^{env}(r) \qquad \textbf{Eqn. S3}$$

The maximum of W delivers the optimal embedding potential, with convergence reached when the sum of the embedded densities reproduces the full system density:

$$\rho^{full}(r) = \rho^{cl}(r) + \rho^{env}(r) \qquad \textbf{Eqn. S4}$$

We carved out a Cu_{17} cluster from a pristine one-sided (1S) model (a 100-atom four-layer (5×5) slab) to represent the active site, where we include six subsurface Cu atoms and 11 surface Cu atoms to create the cluster. The remaining 83 Cu atoms served as the environment. We used an odd-numbered-Cu-atom cluster to maintain an overall even number of electrons when combined with the adsorbates (the H_3O species yields the excess electron for an overall closed-shell singlet solution). We optimized V_{emb} in real-space for the periodic slabs using VASP v. 6.4.1 compiled with a DFET subroutine.[3, 4] We used the same V_{emb}we used for the Cu_{17} cluster in ref. 5.

S2. ECASSCF and ECASPT2 calculations. To generate the reference wavefunctions from ECASSCF, we determined the size and orbital character of the active space (AS) by considering the number of valence electrons of the reaction products and the bonds they form with the Cu surface. As in ref. 5, we employ a combination of "creeping" and "orbital merging" to generate initial guess orbitals.[6] First, we displace the adsorbates from the Cu_{17} cluster by 10 Å for all images along the reaction paths. We then optimized the AS for the desorbed product and "creep" by using the optimized orbitals of an image as the initial guess orbital of the next image. We performed this procedure image by image (with a V_{emb}-free, i.e., bare cluster) along the reverse of the reaction path, all the way to the reactant. After obtaining an optimized set of CASSCF orbitals from the displaced adsorbates for all the structures along the reaction path, we used those orbitals to initialize the ECASSCF calculation for the combined adsorbates and Cu_{17} cluster (i.e., adsorbates are placed on the Cu_{17} cluster as determined in the periodic DFT simulations) – "orbital merging" for the entire reaction. We re-optimized the orbitals with the cluster and adsorbates that are then subject to V_{emb}. **Figs. S4** and **S5** show the final ECASSCF AS(14e,14o) natural orbitals with occupation numbers for the coupling and hydrogenation reactions, respectively. Using the wavefunctions from ECASSCF, we performed ECASPT2 calculations, with an ionization potential-electron affinity (IP-EA) shift[7] of 0.25 Ha and a level-shift correction[8] of 0.30 Ha to aid convergence.

S3. Potential dependence of free energy of reaction and activation. Ref. 9 showed that free energies at a constant number of electrons, obtained from electronic structure calculations, may be converted to free energies at a constant electron potential corresponding to an electrochemical cell (equivalent to a simple Legendre transform). Ref. 9 showed that for the electrochemical reaction $a \rightarrow b$

$$\Delta G_{a\rightarrow b}(\Phi_a) = G_b(\Phi_b) - G_a(\Phi_a) - \frac{(n_e[b] - n_e[a])(\Phi_b - \Phi_a)}{2e} \qquad \textbf{Eqn. S5}$$

likewise,

$$\Delta G_{a\rightarrow b}(\Phi_b) = G_b(\Phi_b) - G_a(\Phi_a) + \frac{(n_e[b] - n_e[a])(\Phi_b - \Phi_a)}{2e} \qquad \textbf{Eqn. S6}$$

It follows that

$$\Delta G_{a\rightarrow b}(\Phi_b) - \Delta G_{a\rightarrow b}(\Phi_a) = \frac{(n_e[b] - n_e[a])(\Phi_b - \Phi_a)}{e} \qquad \textbf{Eqn. S7}$$

and

$$\frac{\Delta G_{a\rightarrow b}(\Phi_b) - \Delta G_{a\rightarrow b}(\Phi_a)}{(\Phi_b - \Phi_a)/e} = (n_e[b] - n_e[a]) = \Delta n_e[a \rightarrow b] \qquad \textbf{Eqn. S8}$$

Assuming **Eqn. S8** holds for a given reaction and a wide range of $\Delta\Phi$, i.e., $\frac{\partial \Delta G}{\partial \Delta \Phi} \approx$ constant (shown to be valid in ref. 9, see Fig. 3 there), then for any arbitrary Φ, $\Delta G_{a\rightarrow b}(\Phi)$ is given by

$$\Delta G_{a\rightarrow b}(\Phi) = \Delta n_e[a \rightarrow b]\frac{(\Phi - \Phi_a)}{e} + \Delta G_{a\rightarrow b}(\Phi_a) \qquad \textbf{Eqn. S9}$$

Substituting **Eqn. S5** into **Eqn. S9**

$$\Delta G_{a\rightarrow b}(\Phi) = \Delta n_e[a \rightarrow b]\frac{(\Phi - \Phi_a)}{e} + G_b(\Phi_b) - G_a(\Phi_a) - \frac{(n_e[b] - n_e[a])(\Phi_b - \Phi_a)}{2e} \qquad \textbf{Eqn. S10}$$

Simplifying **Eqn. S10** yields

$$\Delta G_{a\rightarrow b}(\Phi) = \Delta n_e[a \rightarrow b]\frac{(\Phi - \Phi_a)}{e} + G_b(\Phi_b) - G_a(\Phi_a) - \Delta n_e[a \rightarrow b]\frac{(\Phi_b - \Phi_a)}{2e} \qquad \textbf{Eqn. S11}$$

Finally,

$$\Delta G_{a\to b}(\Phi) = \Delta n_e[a\to b]\frac{\Phi}{e} + [G_b(\Phi_b) - G_a(\Phi_a)] - \Delta n_e[a\to b]\frac{(\Phi_b + \Phi_a)}{2e} \quad \textbf{Eqn. S12}$$

Or simply,

$$\Delta G_{a\to b}(\Phi) = \Delta n_e[a\to b]\frac{\Phi}{e} + \Delta G_{a\to b}(\Phi = 0) \quad \textbf{Eqn. S13}$$

where **Eqn. S13** is a linear function with a slope of $\Delta n_e[a\to b]$ and value at $\Phi = 0$ of

$$\Delta G_{a\to b}(\Phi = 0) = [G_b(\Phi_b) - G_a(\Phi_a)] - \Delta n_e[a\to b]\frac{(\Phi_b + \Phi_a)}{2e} \quad \textbf{Eqn. S14}$$

which is the free energy change at constant number of electrons shifted by the negative of the number of electrons transferred multiplied by the average of the work functions of the initial (a) and final (b) states.

Because we use a *continuum solvent* here, the *work functions we obtain are relative to the bulk continuum solvent potential* (**Table S4**) and not to the vacuum potential. **Section S5** discusses how we calculated for the *work function of the bulk solvent* (Φ_{solv}). We then *add Φ_{solv} to the calculated work functions in continuum solvation to get Φs* (**Table S7**).

Knowing the absolute potential of the RHE or $\mathrm{U_{RHE}}$, we can also reference the Φs to the RHE. Assuming that the calculated work functions are directly comparable to experiments, one may use experimental values for $\mathrm{U_{RHE}}$, which is at pH = 0, simply equal to U of the standard hydrogen electrode ($\mathrm{U_{SHE}}$), i.e., either 4.44 V from Trasatti[10] or 4.281 V from a more recent analysis from Isse and Gennaro.[11] $\mathrm{U_{RHE}}$ is related to $\mathrm{U_{SHE}}$ by the formula

$$\mathrm{U_{RHE}} = \mathrm{U_{SHE}} - 0.0592 \times \mathrm{pH} \quad \textbf{Eqn. S15}$$

S4. Number of electrons transferred. We calculated the number of electrons transferred (Δn_e) at the transition state and products by simple electron counting using the Bader charge partitioning scheme.[12, 13] For example, for the reaction (**R2** in the main text):

$$2^*\mathrm{CO} + \mathrm{Cu_4} + e^- + \mathrm{H^+} \rightarrow [\mathrm{HOC^*{-}^*CO}]^{\delta-} + \mathrm{Cu_4}^{\delta+}$$

the active species is defined as the 2*CO molecules and the four Cu atoms at the active site. *Including the Cu atoms that the active molecules are directly bound to as part of the active species avoids the local charge transfer between them (denoted as δ charge) from being counted as an electrochemical charge transfer.* Upon protonation, the total number of adsorbate electrons now also accounts for the transferred H. The remaining atoms (not including the solvated cation layer, i.e., $\mathrm{Cu_{142}} + 4(\mathrm{H_2O^*})$) constitute the electron bath. In an idealized picture, $\Delta n_e = 1$ after an electron transfer (ET) and a concerted ET and proton transfer (PT) step. In practice, using the above-described partitioning, Δn_e of the active species is given by

$$\Delta n_e[a\to b] = \frac{2n_e[(\mathrm{OC^*{-}^*COH}) + \mathrm{Cu_4}] - 2n_e[2^*\mathrm{CO} + \mathrm{Cu_4}]}{2} \quad \textbf{Eqn. S16}$$

Here, all the molecules are doubled because the reaction is occurring on both sides of the slab model. Accordingly, the overall expression is divided by 2. Δn_e measures the actual number of electrons transferred in the chemical equation above. **Table S5** shows that a near ideal value is achieved only after the full reaction, i.e., upon the formation of $[\mathrm{HOC^*{-}^*CO}]^{\delta-}$. Upon formation of the intermediate $[\mathrm{OC^*{-}^*CO}]^{(1+\delta)-}$ species, only about half an electron was transferred to the molecule. **Table S5** also shows that this definition of transferred charge can also identify ET reactions not concurrently occurring with a PT.

We used an alternative scheme in refs. 14, 15, which is based on ref. 9, where one calculates the amount of electrons transferred in an electro-active reaction by calculating the change of the net charge of the slab and the adsorbed species before and after PCET. The analogous equation for the reaction above is

$$\Delta n'_e[a \rightarrow b] = \frac{q[\mathrm{Cu}_{150} + 2(\mathrm{OC}^*{-}^*\mathrm{COH}) + 4(\mathrm{H_2O}^*)] - q[\mathrm{Cu}_{150} + 4^*\mathrm{CO} + 4(\mathrm{H_2O}^*)]}{2} \quad \textbf{Eqn. S17}$$

where $q = Z - n_e$ and Z is the total valence nuclear charge and n_e is the number of valence electrons, where in this example we have a 5×5 six-layer slab of Cu (i.e., 150 Cu atoms). We distinguish this definition of number of ETs from the one prior by using a different symbol, i.e., $\Delta n'_e$. In **Eqn. S17**, we also include the four H_2O molecules that are directly adsorbed on the Cu slab (two on each side). Note that **Eqn. S17** seems to suggest that an INCREASE in the net charge *q* after the reaction manifests as an electron transfer, which is rather counterintuitive. *It does illustrate the physical meaning of this definition of charge transfer for a capacitor model where one plate is a first Helmholtz layer composed of solvated protons and the other plate is an electrode with excess electrons that is equal in number to the protons. The act of moving the proton from the Helmholtz layer onto the electrode (adding a proton (or any cation) to the surface) is an indirect measure of the assumed concurrent electron transfer to the adsorption site of the displaced proton – a definition that holds true for concerted ET-PT and thus also highlights its limitation.* In ref. 9, the reaction of interest was the hydrogen evolution reaction on Pt(111) where the Volmer and Heyrovsky reactions are guaranteed to be concerted ET-PT. We calculated $q[\mathrm{Cu}_{150} + 2(\mathrm{OC}^*{-}^*\mathrm{COH}) + 4(\mathrm{H_2O}^*)] = -0.112\ e$ and $q[\mathrm{Cu}_{150} + 4^*\mathrm{CO} + 4(\mathrm{H_2O}^*)] = -2.042\ e$, which yields $\Delta n'_e[a \rightarrow b] = (1.930/2)\ e = 0.965\ e$. The $q[\mathrm{Cu}_{150} + 4^*\mathrm{CO} + 4(\mathrm{H_2O}^*)]$ being ~ $-2\ e$ shows that in our models, the two hydronium ions fully ionize, giving one electron to the slab and adsorbates per cation.

$\Delta n_e[a \rightarrow b]$ is a direct measure of ET, whereas $\Delta n'_e[a \rightarrow b]$ is an indirect measure of ET via the movement of the positive charge. To relate the two methods of quantifying charge transfer, we break down **Eqn. S17**, substituting $Z - n_e$ for q and partitioning the system into the environment and the cluster, to write:

$$\Delta n'_e[a \rightarrow b] = \frac{\Delta Z - \Delta n_e[\mathrm{Cu}_{142} + 4(\mathrm{H_2O}^*)] - 2n_e[(\mathrm{OC}^*{-}^*\mathrm{COH}) + \mathrm{Cu}_4] + 2n_e[2^*\mathrm{CO} + \mathrm{Cu}_4]}{2} \quad \textbf{Eqn. S18}$$

Note that here $\Delta Z = 2$ (one per proton transferred), therefore after further simplification **Eqn. S18** becomes

$$\Delta n'_e[a \rightarrow b] = 1 - \frac{\Delta n_e[\mathrm{Cu}_{142} + 4(\mathrm{H_2O}^*)]}{2} - n_e[(\mathrm{OC}^*{-}^*\mathrm{COH}) + \mathrm{Cu}_4] + n_e[2^*\mathrm{CO} + \mathrm{Cu}_4] \quad \textbf{Eqn. S19}$$

using the expression for $\Delta n_e[a \rightarrow b]$ (**Eqn. S16**) into **Eqn. S19** yields

$$\Delta n'_e[a \rightarrow b] = 1 - \left\{\frac{\Delta n_e[\mathrm{Cu}_{142} + 4(\mathrm{H_2O}^*)]}{2} + \Delta n_e[a \rightarrow b]\right\} \quad \textbf{Eqn. S20}$$

The term

$$-\left\{\frac{\Delta n_e[\mathrm{Cu}_{142} + 4(\mathrm{H_2O}^*)]}{2} + \Delta n_e[a \rightarrow b]\right\} \quad \textbf{Eqn. S21}$$

is the negative of the combined electron gain of the slab and adsorbates and is simply equivalent to the loss of electrons in the water cluster layer (first Helmholtz layer) after the reaction, which is ideally 0. *This definition of charge transfer therefore assumes first a full electron transfer then subtracts the number of electrons GAINED by the system (i.e., lost by the solvent layer) after the reaction*, which is again rather counter intuitive. This behavior however again reflects the physical meaning of this ET expression, as stated above: *it is as a measure of transfer of positive charge, which the method assumes must be matched, in equal magnitude, by electron transfer*.

It is now clear that $\Delta n'_e[a \rightarrow b]$ may not aptly describe an ET without an explicit and concurrent or simultaneous PT. The advantage of this definition, however, is that one only must define which atoms belong to the surface and which belong to the explicit solvent (water cluster) layer. This scheme thus avoids an arbitrary definition of the active species, except at the transition state, where one must decide whether the H already is or is not part of the surface. If the proton is already part of the adsorbate, then ET will be closer to 1, if not, then ET is closer to 0. Regardless of the merits and limitations of $\Delta n_e[a \rightarrow b]$ and $\Delta n'_e[a \rightarrow b]$, the two definitions are quantitatively similar for concerted ET-PT as we show in **Table S5**.

Accordingly, for the reaction (**R3** in the main text)

$$^{*}CO + e^{-} + H^{+} \rightarrow {}^{*}COH$$

we define Δn_e as

$$\Delta n_e[a \rightarrow b] = \frac{2n_e[\mathrm{COH}] - 2n_e[\mathrm{CO}]}{2} \quad \textbf{Eqn. S22}$$

In **Eqn. S22**, we did not include any of the Cu atoms to which *COH is adsorbed, because the interaction of *COH with the surrounding Cu should be covalent, unlike in $[HOC^{*}–{}^{*}CO]^{\delta-}$ and $[OC^{*}–{}^{*}CO]^{(1+\delta)-}$ where additional local partial charge transfer (δ) was needed to explain the structure of the adsorbates. We calculated a value slightly above 1 (1.2 *e*) using **Eqn. S22** for the hydrogenation reaction as shown in **Table S5**, which is similar in magnitude to $[HOC^{*}–{}^{*}CO]^{\delta-}$. The value is similar but not equivalent to the old definition (expression analogous to **Eqn. S17**) of charge transfer (0.95 *e*), which is also presented in **Table S5**. When we include the four Cu atoms at the reactive site in **Eqn. S22**, we get a value (~0.7 *e*) that is far from the old definition, but closer to what was calculated in ref. 14 that was calculated using the old definition, i.e., $\Delta n'_e[a \rightarrow b]$ (an analogous expression for **Eqn. S17** for this reaction) and was evaluated in vacuum, whereas here we evaluate the Bader charges in continuum solvent.

S5. Value of Φ_{solv}. One can independently solve for Φ_{solv} within the same level of theory and atomic models to establish a consistent reference absolute potential. We used a scheme that invokes the computational hydrogen electrode (CHE) method. To accomplish this, here we calculated the reaction free energy of the overall reaction:

$$2^{*}CO + Cu_4 + e^{-} + H^{+} \rightarrow [HOC^{*}–{}^{*}CO]^{\delta-} + Cu_4^{\delta+}$$

using the CHE

$$e^{-} + H^{+} \leftrightarrow ½\, H_2$$

as a reference ($\Delta G_{a\rightarrow b}(\mathrm{CHE})$) from DFT-PBE+D3BJ. Hence, we removed an H from each of the hydronium ions in the reactant (leaving the four H_2O molecules) to generate a reactant (relaxing the resulting structure) in which there is no excess charge in the solution nor the slab. We then use half the free energy of a hydrogen gas (H_2) as the free energy of an electron-proton pair in solution.

Without loss of generality, we can replace all Φs in the equations in **section S3** with $\Phi - \Phi_{\mathrm{solv}}$.[16] Because $\Delta G_{a\rightarrow b}(\mathrm{CHE\ at\ pH} = 0)$ corresponds to the free energy at 0 V vs. $\mathrm{U_{SHE}}$, then $\Phi - \Phi_{\mathrm{solv}} = e\mathrm{U_{SHE}} - \Phi_{\mathrm{solv}}$ when $\Delta G_{a\rightarrow b}(\Phi - \Phi_{\mathrm{solv}}) = \Delta G_{a\rightarrow b}(\mathrm{CHE\ at\ pH} = 0)$, which we calculated within DFT-PBE+D3BJ. After solving for Φ in **Eqn. S13** and then substituting $\Delta G_{a\rightarrow b}(\mathrm{CHE\ at\ pH} = 0)$ for $\Delta G_{a\rightarrow b}(\Phi)$ and $\Phi - \Phi_{\mathrm{solv}}$ for Φ, we obtain

$$\Phi - \Phi_{\mathrm{solv}} = \frac{\Delta G_{a\rightarrow b}(\mathrm{CHE\ at\ pH} = 0) - \Delta G_{a\rightarrow b}(\Phi - \Phi_{\mathrm{solv}} = 0)}{\Delta n_e[a \rightarrow b]/e} = e\mathrm{U_{SHE}} - \Phi_{\mathrm{solv}} \quad \textbf{Eqn. S23}$$

Using **Eqn. S14**, one can calculate

$$\Delta G_{a\rightarrow b}(\Phi - \Phi_{\mathrm{solv}} = 0) = [G_b(\Phi_b - \Phi_{\mathrm{solv}}) - G_a(\Phi_a - \Phi_{\mathrm{solv}})] - \Delta n_e[a \rightarrow b]\left[\frac{(\Phi_b + \Phi_a)}{2e} - \frac{\Phi_{\mathrm{solv}}}{e}\right] \quad \textbf{Eqn. S24}$$

To evaluate **Eqn. S23**, we evaluated first **Eqn. S24** using the PCET reaction free energy at constant number of electrons ($[G_b(\Phi_b - \Phi_{\mathrm{solv}}) - G_a(\Phi_a - \Phi_{\mathrm{solv}})]$), the number of electrons transferred ($\Delta n_e[a \rightarrow b]$), and work functions relative to the solvent ($\Phi_a - \Phi_{\mathrm{solv}}$ and $\Phi_b - \Phi_{\mathrm{solv}}$) - **Tables S3-S5**. After computing $\Delta G_{a\rightarrow b}(\Phi - \Phi_{\mathrm{solv}} = 0)$ and $\Delta G_{a\rightarrow b}(\mathrm{CHE\ at\ pH} = 0)$ in **Table S6**, we evaluated **Eqn. S23**. We find $e\mathrm{U_{SHE}} - \Phi_{\mathrm{solv}} = 3.908$ eV. From the most accurate experimental value of $\mathrm{U_{SHE}} = 4.281$ V,[11] we derived a semi-empirical $\Phi_{\mathrm{solv}} = +0.373$ eV. Note that the hydronium ion is near the electrode surface (first Helmholtz layer) in the explicit hydronium model, whereas this cation is, by definition, in bulk solution within the CHE framework. *Φ_{solv} as defined in **Eqn. S23** therefore represents more than the work function of the solvent model but also the necessary energy shift (by means of a potential shift) associated with free energy needed to move the hydronium from the bulk solution into the first Helmholtz layer.*

We performed the same calculation for the hydrogenation of a *CO to *COH using a similar model. Using the same expression above (**Eqn. S23**) and again using DFT-PBE+D3BJ free energetics, we calculated to be $eU_{\mathrm{SHE}} - \Phi_{\mathrm{solv}} = 3.878$ eV and thus a value of $\Phi_{\mathrm{solv}} = +0.403$ eV. By calculating a separate Φ_{solv} for each reaction type, we achieve model consistency that may benefit from error cancelation while still enabling direct comparison of energetics within the same absolute potential scale while keeping the same reference potential value as we have done in **Fig. 2** in the main text. **Table S6** summarizes the CHE-referenced reaction free energies and Φ_{solv} for both reactions. **Table S7** summarizes the resulting Φs using the $(\Phi - \Phi_{\mathrm{solv}})$s (**Table S4**) and calculated Φ_{solv}s (**Table S6**).

Supplementary Note 1

CO reduction to *COH via PCET*. We follow the same procedure we used in the electrochemical coupling of *CO. We started with a *CO adsorbed atop a Cu atom and introduced an Eigen cation ($(H_2O)_3(H_3O)^+$) subject to an implicit solvent to simulate hydrogenation to *COH adsorbed at a hollow site. We mapped out the minimum energy path for this reaction using a two-sided slab model (the same used for the coupling reaction) using CI-NEB.[17] **Fig. S2 shows select structures along the reaction path and energetics in panels **A** and **B**, respectively. The pathway is similar to the one found by Zhao et al. in ref. 14 with a one-sided slab model, but we extended the reaction path such that we find a local minimum with DFT for the product, which was not considered in ref. 14 (the difference being a bond rotation, described next). As the reaction proceeds, *CO first migrates to an adjacent hollow site, where the C-O bond stretches from a triple (1.16 Å) to a double (1.23 Å) bond. At this point, one of the H_2O molecules solvating the hydronium ion forms a hydrogen bond with *CO. This process is energetically downhill within DFT (-0.29 eV) but uphill within ECASPT2 (+0.53 eV). This step is followed by the shuttling of the proton from H_3O^+ to *CO via the hydrogen-bonded H_2O, going through an inflection point structure where *COH is now a hydrogen-bond donor to the H_2O molecule that shuttled the proton through: **Fig. S2A**, structure labelled hollow (inflection point). From **Fig. S2B**, one can see that this structure is not a proper minimum in either DFT (marked by a purple circle) or ECASPT2 (although considered as product in ref. 14). To prevent the proton from hopping back to the water cluster (energetically downhill), the OH group can rotate away so that the hydrogen-bond distance increases to 2.6 Å at the transition state. The OH can rotate further until it become a hydrogen-bond acceptor to the same H_2O molecule, which is now a local minimum. In **Fig. S2**, we show in **A** and mark in **B** the hollow *COH structure to the right of the transition state that is lower in energy within ECASPT2. We calculated a barrier ($E^‡$) of 0.72 and 1.01 eV within DFT and ECASPT2 relative to the atop *CO, respectively. Interestingly, if the barrier is measured relative to the hollow *CO within DFT, then the barrier will also be 1.01 eV. This illustrates that error cancelation can lead to fortuitously correct quantitative prediction despite the incorrect qualitative predictions, i.e. atop vs. hollow (the reverse behavior is usually assumed). Also rather coincidentally, although ref. 14 used the inflection point structure as the product, leading to a lower DFT $G^‡$ = 0.11 vs. 0.75 eV here (**Table S3**), the calculated ECASPT2 $G^‡$ therein has smaller deviation from what is calculated here: 0.75 vs. 1.04 eV (**Table S3**).

Supplementary Figures

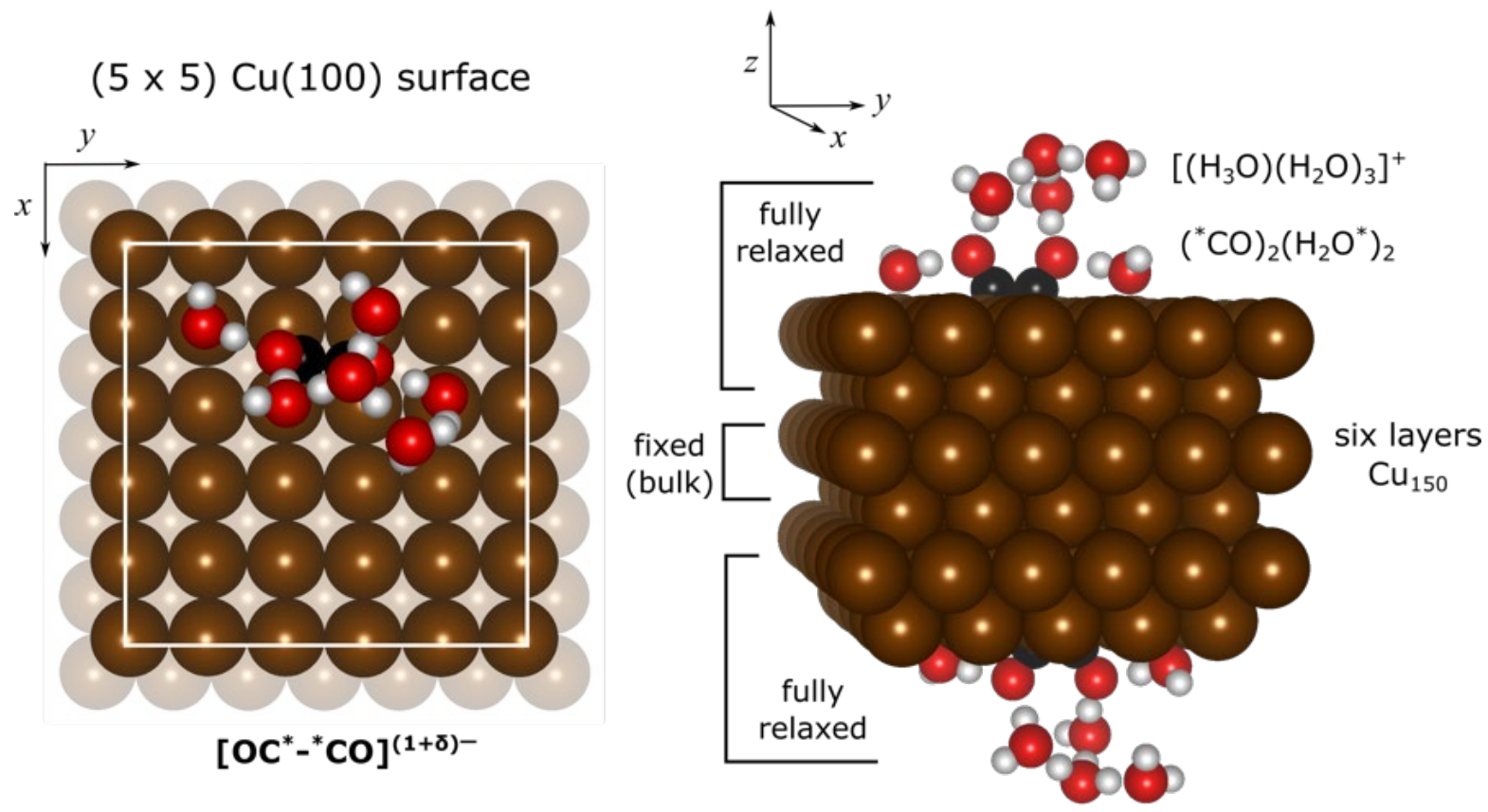

Fig. S1. Simulation models. Supercell (left: top, right: side view) showing a six-layer (5×5) supercell Cu(100) laterally periodic slab. OC*–*CO with C atoms attached to the bridge sites of the surface, two adsorbed water molecules, and an Eigen cation on each side of the slab are shown. Cu – dark brown spheres, C – black, O – red, and H – white. Vectors: $x = [011]$, $y = [0\bar{1}1]$, $z = [100]$. White box on the left marks the boundaries of the (5×5) repeat unit.

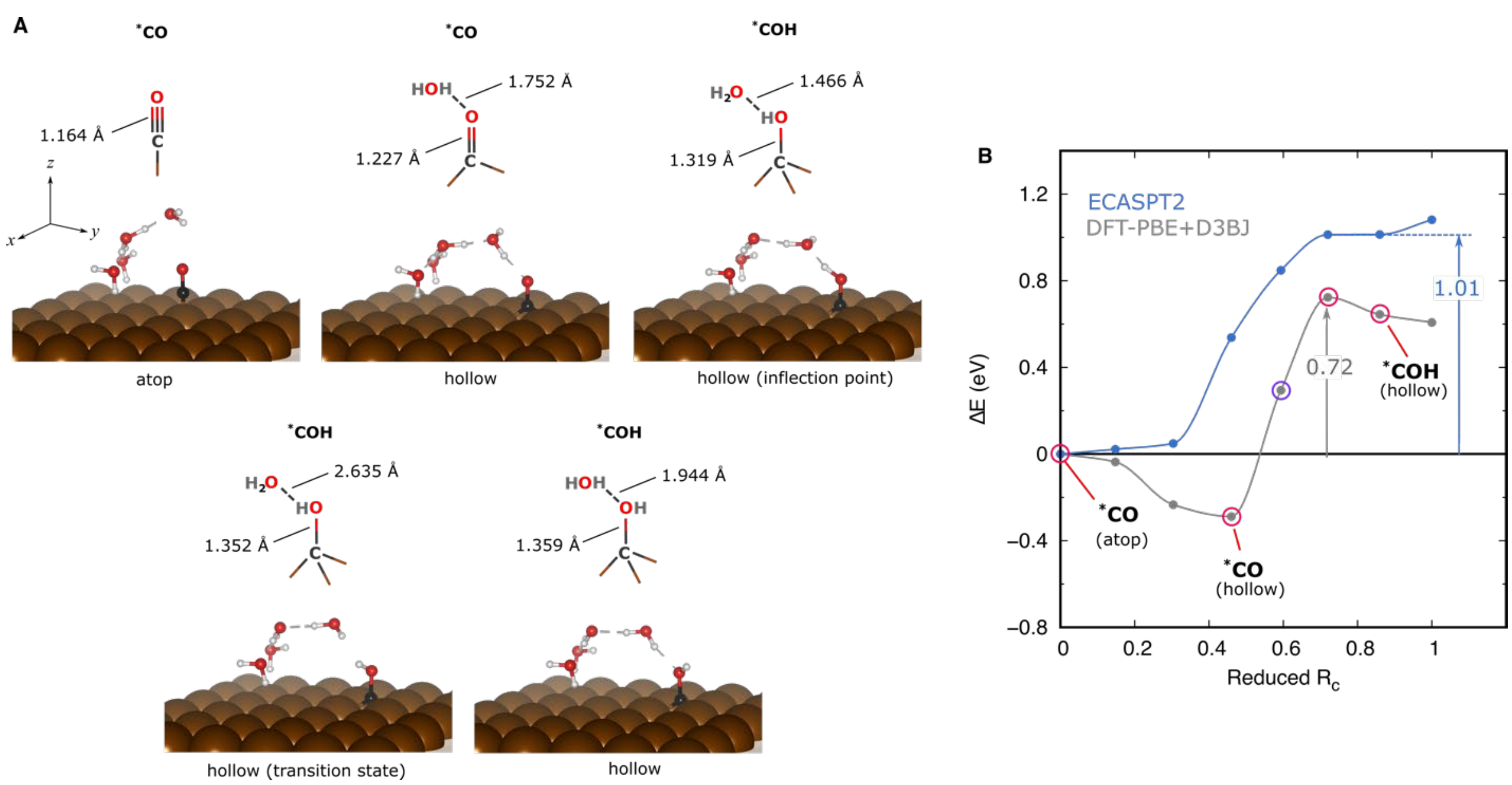

Fig. S2. A, DFT-PBE+D3BJ solvent-optimized structures along the minimum-energy coupling path showing atop *CO (reactant), hollow *CO (a DFT minimum), hollow *CO (an inflection point designated as product in ref. 14), hollow *COH as hydrogen bond donor (transition state), and hollow *COH as hydrogen bond acceptor (product). The C-O and hydrogen bond lengths are annotated in their corresponding Lewis structures. Cu – dark brown spheres, C – black, O – red, and H – white. Vectors: $x = [011]$, $y = [0\bar{1}1]$, $z = [100]$. **B**, *CO to *COH energy curves calculated using DFT-PBE-D3BJ (grey) and ECASPT2 (light blue) in continuum water. The corresponding structures for the data points marked with red and purple circles are shown in **A** with the DFT-predicted stationary structures labelled. The purple circle marks the position of the inflection point structure that was considered as the product in ref. 14. The overall reaction barrier and some reaction energies are marked (numbers on the figures are all in eV).

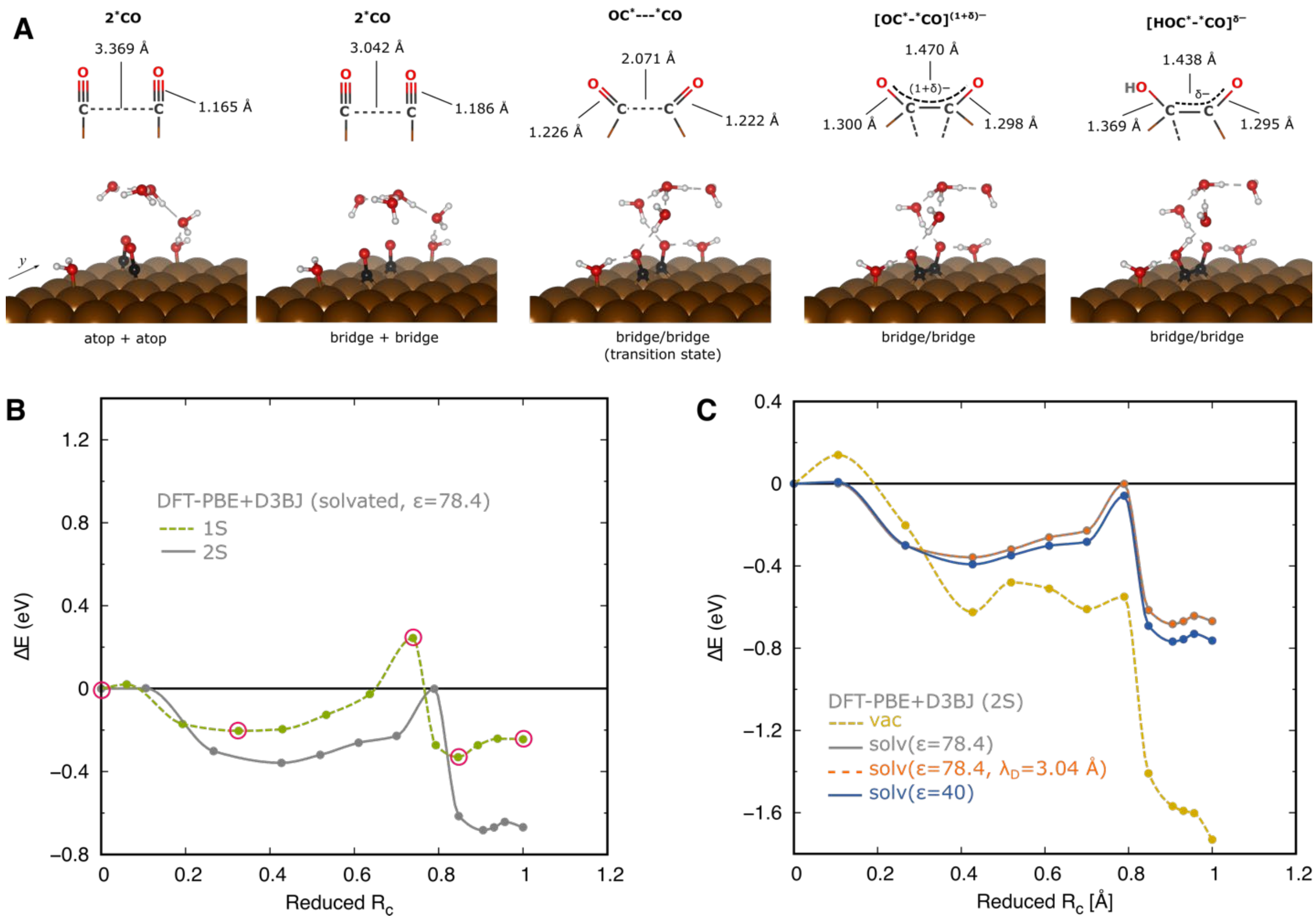

Fig. S3. **A**, DFT-optimized structures along the minimum-energy coupling path when *using the 1S model*, showing atop + atop 2*CO (reactant), bridge + bridge 2*CO (a DFT minimum), transition state, and products: bridge/bridge $[OC^*–{}^*CO]^{(1+\delta)-}$ (first product) and bridge/bridge $[HOC^*–{}^*CO]^{\delta-}$ (second product). Compared to the structures in **Fig.1** of the main text, the effects of using 1S instead of 2S model on the atomic structures appear to be small (within 0.1 Å deviation in the bond lengths). Comparison between the DFT-PBE+D3BJ PECs calculated from: **B**, the 1S and 2S models in continuum water, and **C**, the 2S model in vacuum with dipole correction (vac) and continuum water (solv, dielectric constant ε = 78.4). The reaction energies were normalized per *CO pair. The corresponding structures for the data points marked in **B** with red circles are shown in **A.** In **B**, the 1S model leads to overestimation of the barrier and a less exothermic reaction, differing by 0.24 and 0.42 eV, respectively. In the 1S model, the slab is unphysically polarized by the solvent such that the reactant, where the excess electron remains in the metal, is artificially stabilized. This effect gradually disappears as the electron localizes onto the adsorbate. For the vacuum PEC in **C**, we used the structures optimized in the implicit solvent. In **C**, vacuum cannot screen the charged hydronium ions (despite their explicit solvation by three water molecules in the Eigen cation) which destabilizes the reactant, which leads to a catastrophically incorrect PEC. As the protons (one on each side) transfer onto the surface and the electrons re-localize onto the adsorbates (charge recombination), i.e., as the products form, dramatic stabilization occurs. Both the 2S model and solvation are therefore needed to properly model the electroactive coupling of *CO. Addition of ionic screening (λ_D = 3.04 Å) has a negligible effect: pure water and 1 M monovalent ion aqueous solution are almost perfectly overlapping. Lastly, reduction in the solvent's ε by 50% lowers the transition state and product relative to the reactant by less than 0.1 eV.

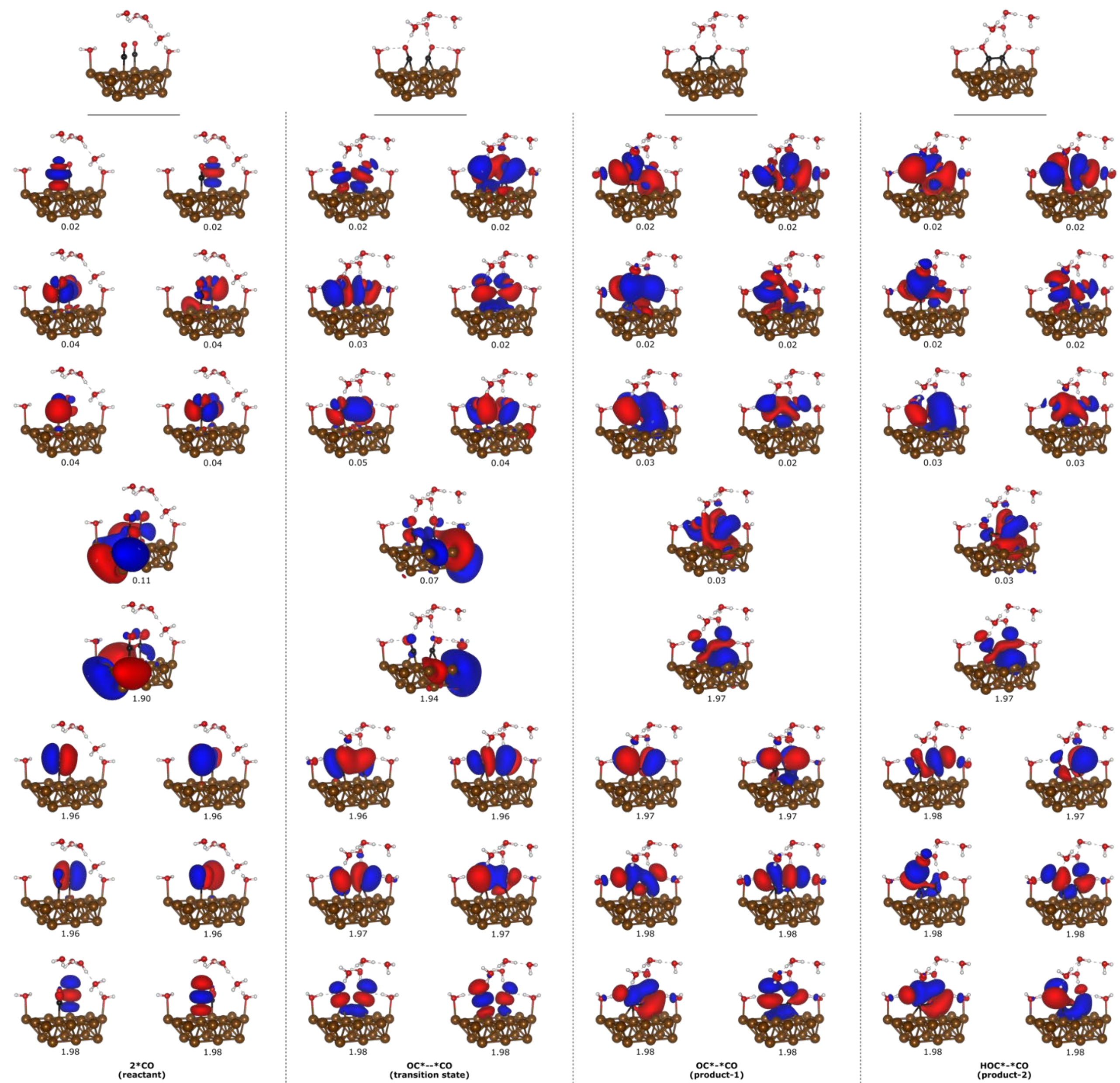

Figure S4. Active space (AS) natural orbitals (with occupation numbers) from ECASSCF AS(14e,14o) for the reactant, transition state, and products along the PCET pathway for the coupling of two *COs to [OC*–*COH]$^{\delta-}$. The AS originated from 12 C-O-derived orbitals and two Cu 4*s*-derived orbitals. On top of each column of orbitals are the corresponding structures without orbitals. We followed the electron counting scheme in ref. 5 to define the size of the active space and used the method described therein to generate initial guess orbitals (see **section S2**).

Fig. S5. Active space (AS) natural orbitals (with occupation numbers) from ECASSCF AS(14e,14o) for the reactant, an intermediate (*CO hollow), transition state, and product along the PCET pathway for *CO to *COH. On top of each column of orbitals are the corresponding structures without orbitals. The AS originated from six C-O-derived orbitals, six O-H-derived orbitals, and two Cu 4*s*-derived orbitals. We use a similar set of AS orbitals as in ref. 14 and used the method described in ref. 5 to generate initial guess orbitals (see **section S2**).

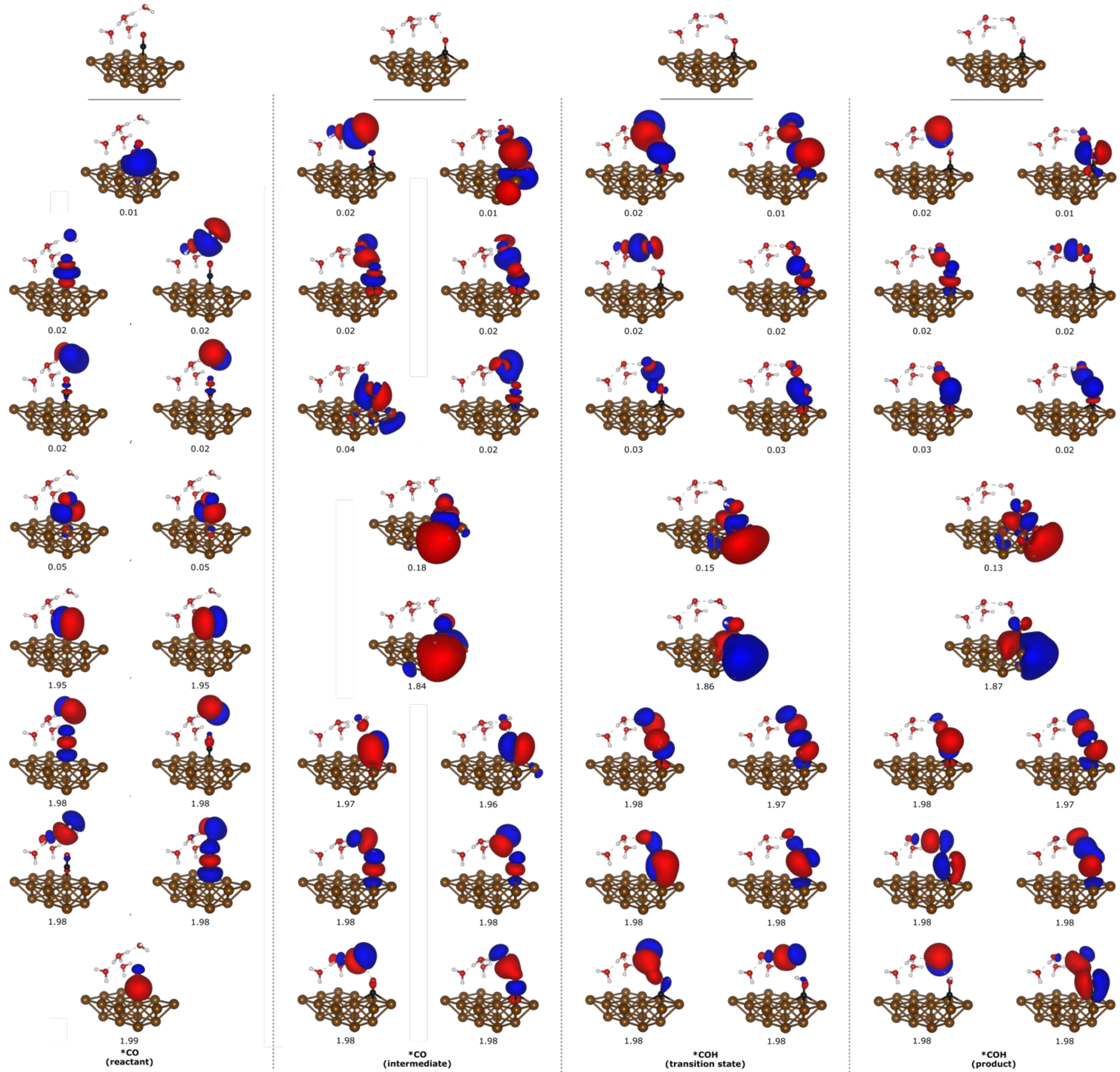

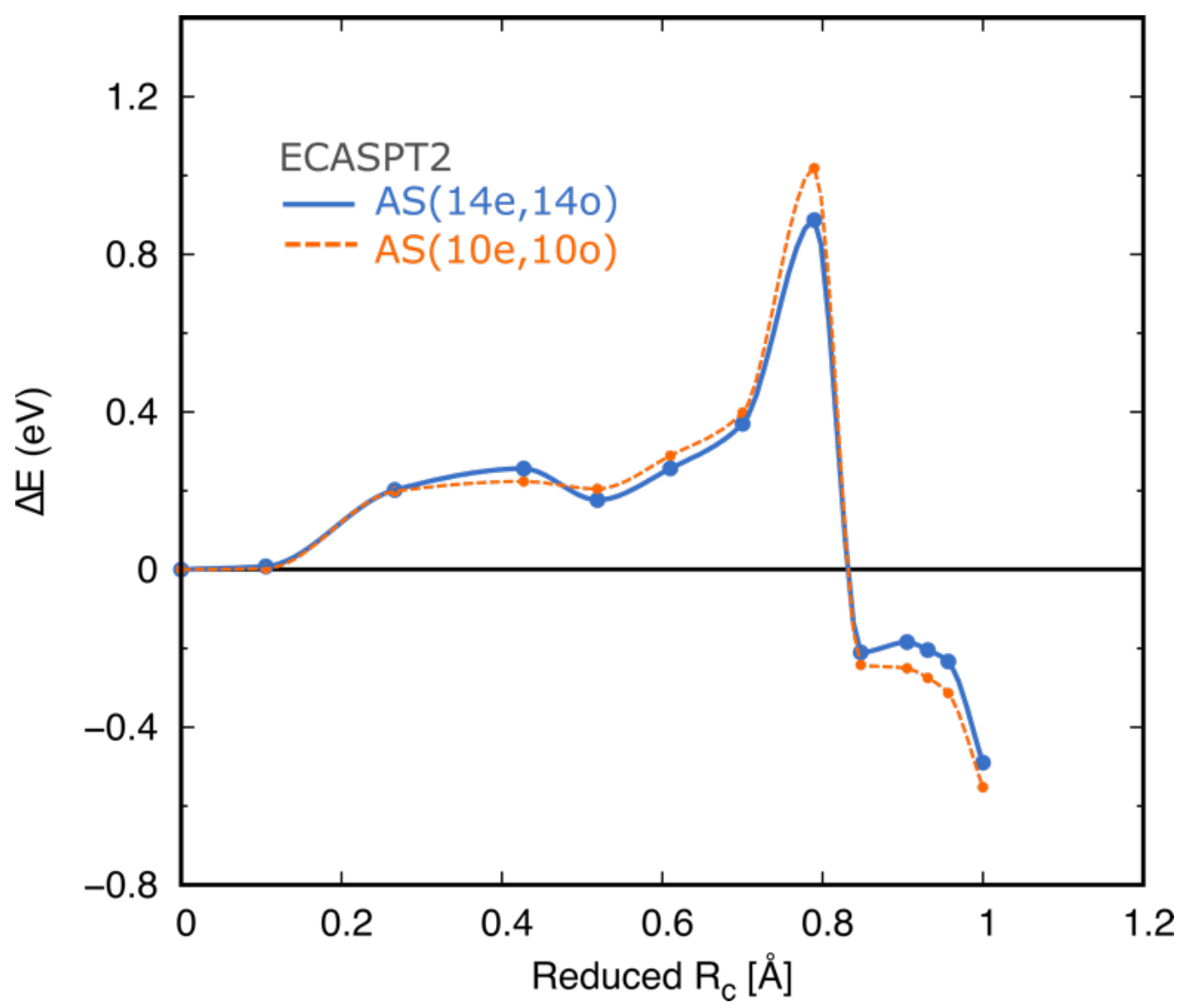

Fig. S6. Sensitivity of ECASPT2 potential energy curve with CASSCF AS size for the reaction 2*CO + Cu_4 + e^- + H^+ → $[HOC^*–^*CO]^{\delta-}$ + $Cu_4^{\delta+}$. Removing two pairs of nearly occupied and nearly empty orbitals from AS(14e,14o) (AS natural orbitals illustrated in Fig. S4) to yield AS(10e,10o) results in the barrier increasing by 0.13 eV and the reaction energy decreasing by 0.06 eV. Energy differences between the two AS sizes are < 0.1 eV, except at the transition state (where correlation is most crucial).

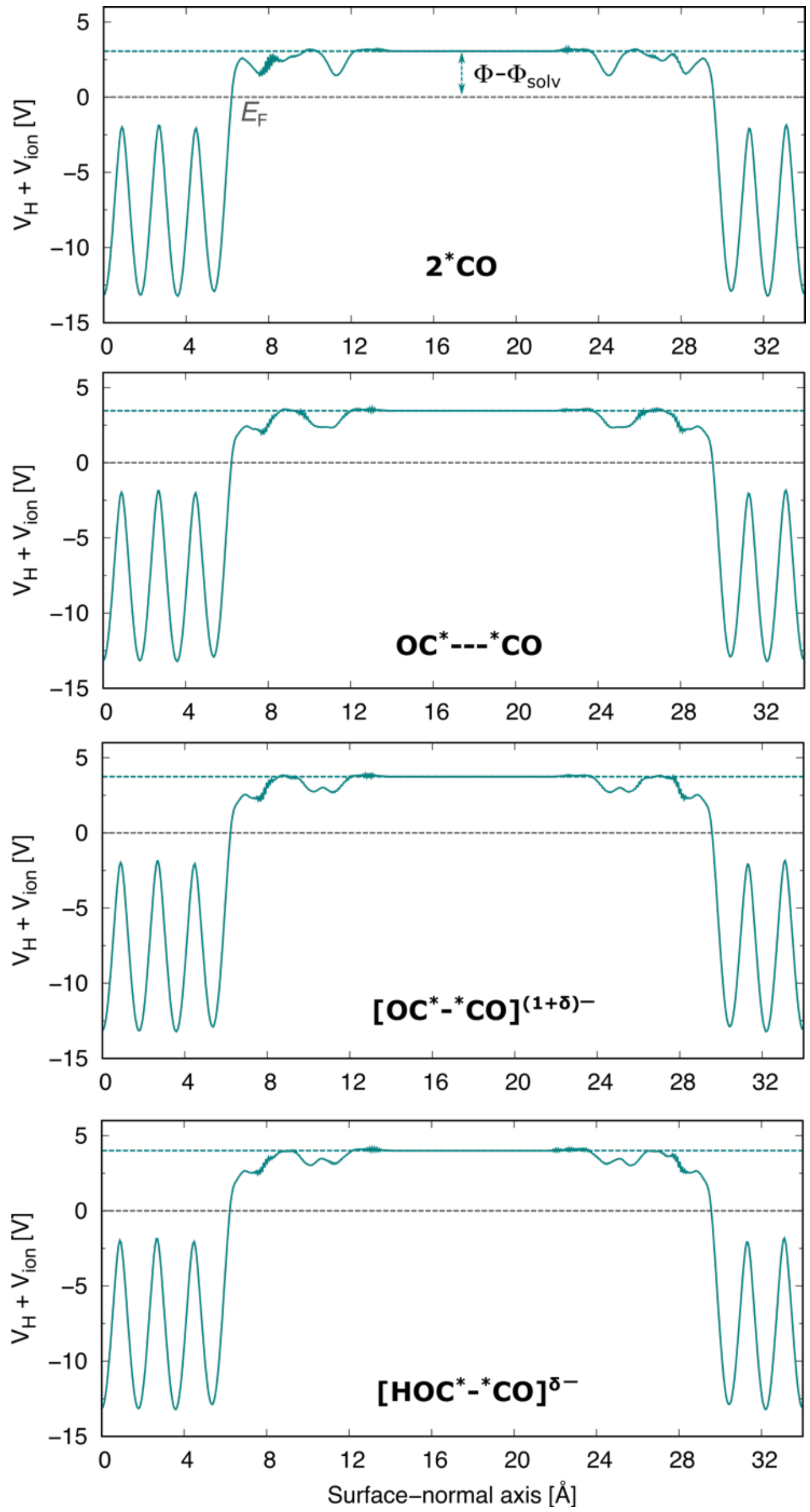

Fig. S7. Electrostatic potential as a function of position along the surface normal of Cu(100) for the stationary and transition state structures of the reaction $2^{*}CO + Cu_4 + e^{-} + H^{+} \rightarrow [HOC^{*}–^{*}CO]^{\delta-} + Cu_4^{\delta+}$. Calculated using the 150-atom six-layer (5×5) supercell 2S model with |c| = 34.0 Å with the adsorbates as labelled (structures shown in **Fig. 1A** of the main text). The potentials were referenced to the Fermi level (E_F).

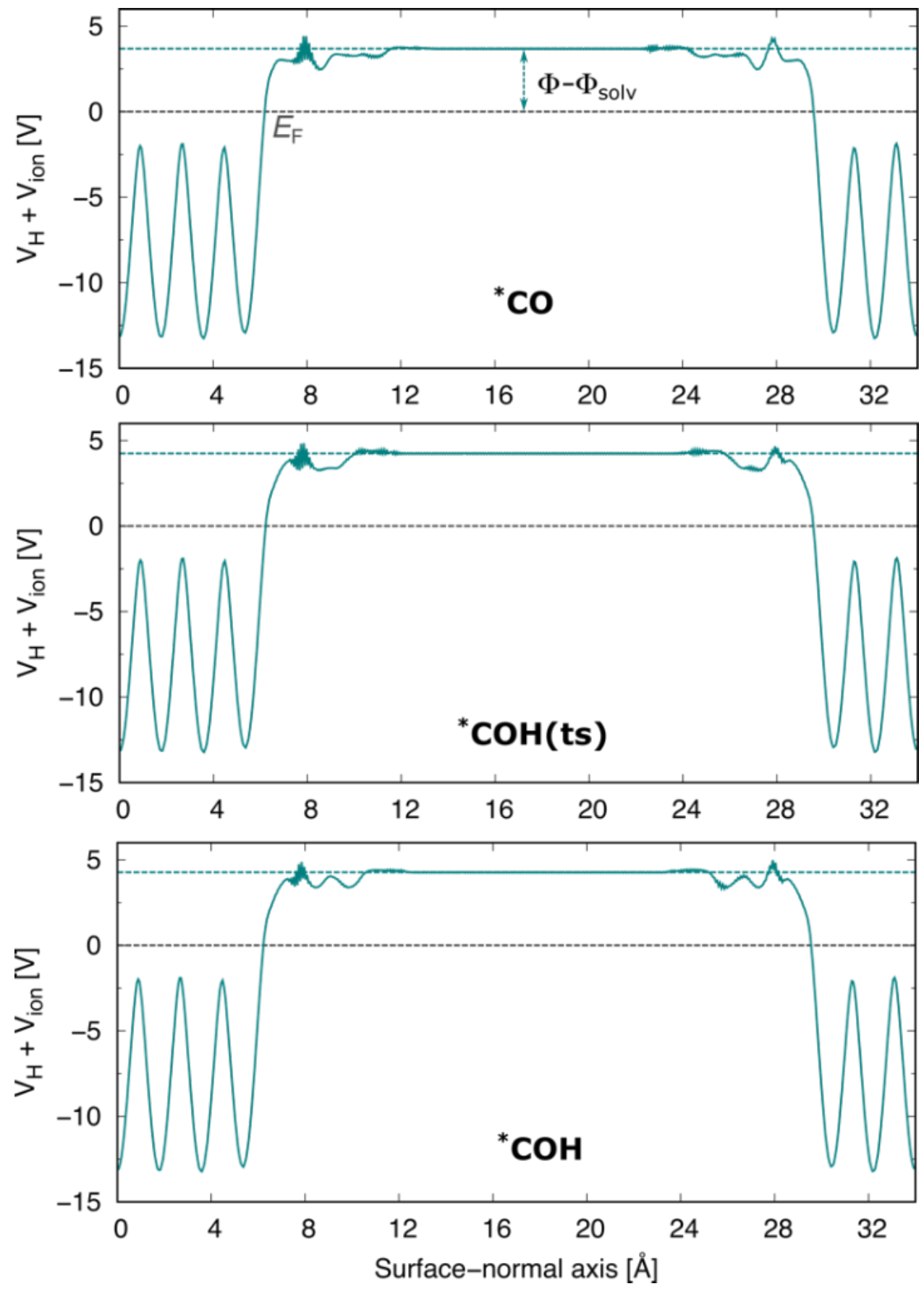

Fig. S8. **Electrostatic potential as a function of position along the surface normal of Cu(100).** Calculated using the 150-atom six-layer (5×5) supercell 2S model with |c| = 34.0 Å with the adsorbates as labelled (structures shown in **Fig. S2A**). The potentials were referenced to the Fermi level (E_F).

Supplementary Tables

Table S1. Equilibrium (0 K) C-C and C-O bond lengths in select C_2 gaseous (oxygenated) hydrocarbons calculated via DFT-PBE+D3BJ, as structural references for C_2 adsorbates (also appears in the SI of ref. 5).

**Molecule**[a]	**Lewis structure**[b]	**C-C bond length [Å]**	**C-O bond length [Å]**
trans-glyoxal	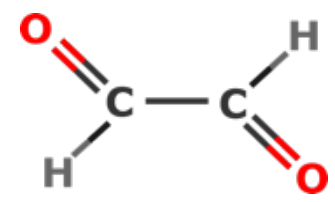	1.527	1.217 (C=O)
trans-glycolaldehyde	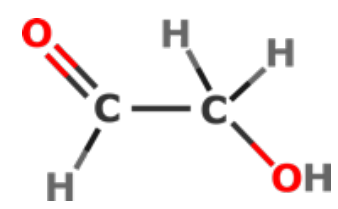	1.515	1.217 (C=O)<br>1.425 (C-OH)
trans-1,2-ethendiol	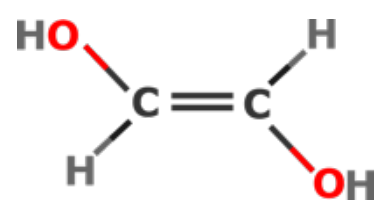	1.338	1.386 (C-OH)
anti-ethylene glycol	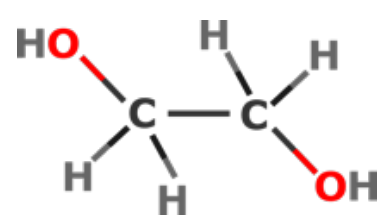	1.520	1.432 (C-OH)
cis-glyoxal	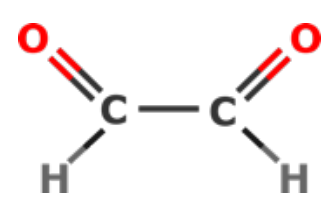	1.546	1.212 (C=O)
cis-glycolaldehyde	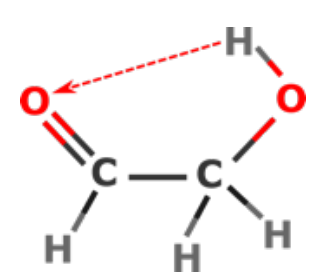	1.503	1.223 (C=O)<br>1.403 (C-OH)
cis-1,2-ethendiol	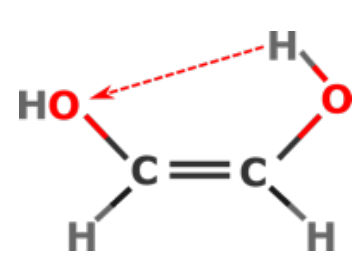	1.340	1.393 (C-OH)<br>1.370 (C-OH)
gauche-ethylene glycol	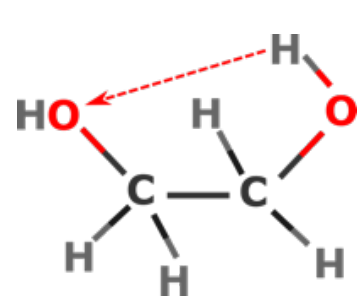	1.516	1.439 (C-OH)<br>1.427 (C-OH)
Ethene	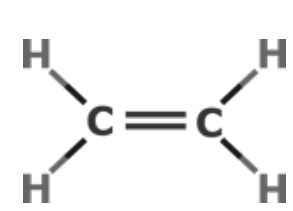	1.333	-
Ethane	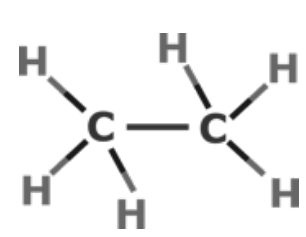	1.529	-

[a]cis and gauche are lower in energy than trans and anti, respectively, except for glyoxal, because cis and gauche enable intramolecular hydrogen bonding when the terminal moieties can do so. The C_2 adsorbates that bond through the C atoms adopt structures similar to the cis and gauche conformers. [b]Intramolecular hydrogen bonding for molecules with OH, if present, is illustrated using red dashed arrows.

Table S2. Reaction energetics (w/o ZPE) in continuum water (ε = 78.4).

Reaction	ΔE_{rxn} [eV][a]		$E^{\ddagger}$ [eV][a]	
	DFT-PBE+D3BJ	ECASPT2	DFT-PBE+D3BJ	ECASPT2
$2^{*}CO + Cu_4 + e^- \rightarrow [OC^{*}–^{*}CO]^{(1+\delta)-} + Cu_4^{\delta+}$	-0.68	-0.18	0.00	0.89
$2^{*}CO + Cu_4 + e^- + H^+ \rightarrow [HOC^{*}–^{*}CO]^{\delta-} + Cu_4^{\delta+}$	-0.67	-0.49	-	-
$CO + e^- + H^+ \rightarrow {}^{*}COH$	0.61	1.01	0.72	1.01

[a]constant no. of electrons

Table S3. Reaction free energetics (constant no. of electrons) in continuum water (ε = 78.4) at 298.15 K.

Reaction	ΔG_{rxn} [eV][a]		$G^{\ddagger}$ [eV][a]	
	DFT-PBE+D3BJ	ECASPT2	DFT-PBE+D3BJ	ECASPT2
$2^{*}CO + Cu_4 + e^- \rightarrow [OC^{*}–^{*}CO]^{(1+\delta)-} + Cu_4^{\delta+}$	-0.58	-0.08	0.05	0.94
$2^{*}CO + Cu_4 + e^- + H^+ \rightarrow [HOC^{*}–^{*}CO]^{\delta-} + Cu_4^{\delta+}$	-0.49	-0.31	-	-
${}^{*}CO + e^- + H^+ \rightarrow {}^{*}COH$	0.63	1.03	0.75	1.04

[a]constant no. of electrons

Table S4. Work function vs. the implicit solvent potential ($\Phi - \Phi_{solv}$) from DFT-PBE+D3BJ for the PCET $2^{*}CO$ coupling and ${}^{*}COH$ formation reactions on the 2S slabs in continuum water (ε = 78.4).[a] R, T, and P refer respectively to reactant, transition state, and product.

Reaction	$\Phi - \Phi_{solv}$ [eV]			$\frac{(\Phi_a+\Phi_b)}{2} - \Phi_{solv}$ [eV]	
	R	T	P	R,T	R,P
$2^{*}CO + Cu_4 + e^- \rightarrow [OC^{*}–^{*}CO]^{(1+\delta)-} + Cu_4^{\delta+}$	3.057	3.452	3.737	3.254	3.396
$2^{*}CO + Cu_4 + e^- + H^+ \rightarrow [HOC^{*}–^{*}CO]^{\delta-} + Cu_4^{\delta+}$	3.057	-	3.988	-	3.522
${}^{*}CO + e^- + H^+ \rightarrow {}^{*}COH$	3.678	4.266	4.290	3.972	3.984

[a]Calculated from electrostatic potential plots in **Figs. S7** and **S8**

Table S5. Number of electrons of the reactive adsorbate (n_e)[a] and number of electrons transferred (Δn_e) per reaction[b] for the PCET $2^{*}CO$ coupling and ${}^{*}COH$ formation reactions using Bader partitioning applied to slab DFT-PBE+D3BJ electron densities in continuum water (ε = 78.4). R, T, and P refer respectively to reactant, transition state, and product.

Reaction	n_e[a] [e]			Δn_e [e]	
	R	T	P	T	P
$2^{*}CO + Cu_4 + e^- \rightarrow [OC^{*}–^{*}CO]^{(1+\delta)-} + Cu_4^{\delta+}$	64.387	64.683	64.981	0.296	0.594
$2^{*}CO + Cu_4 + e^- + H^+ \rightarrow [HOC^{*}–^{*}CO]^{\delta-} + Cu_4^{\delta+}$	64.387	-	65.458	-	1.071 (0.965)[c]
${}^{*}CO + e^- + H^+ \rightarrow {}^{*}COH$	10.227	11.426	11.441	1.199 (0.932)[c]	1.214 (0.952)[c]

[a][2*CO + Cu_4], [$OC^{*}–^{*}CO + Cu_4$], [$HOC^{*}–^{*}CO + Cu_4$], ${}^{*}CO$, or ${}^{*}COH$ per side - see **section S4** (**Eqn. S16** and **S22**)

[b]reaction occurs on both sides of the slab

[c]using scheme from ref. 9 - see **section S4** (**Eqn. S17** and analogous expression for ${}^{*}COH$)

Table S6. DFT-PBE+D3BJ reaction energies vs. CHE (pH = 0, U = 0 V vs SHE) in continuum water (ε = 78.4) and the corresponding Φ_{solv}.[a]

Reaction	ΔE_{rxn} [eV][a]	ΔG_{rxn} [eV][a]	Φ_{solv} [eV]
$2{}^*CO + ½H_2 + Cu_4 \rightarrow [HOC^*–{}^*CO]^{\delta-} + Cu_4^{\delta+}$	-0.48	-0.08	0.373
${}^*CO + ½H_2 \rightarrow {}^*COH$	0.21	0.54	0.403

[a]For H_2(g), we use the tabulated integrated isobaric heat capacity and entropy from the NIST-JANAF database[18] along with the calculated zero-point energy associated with the H-H bond stretch to calculate its free energy at 298.15 K

Table S7. Work function (Φ) from DFT-PBE+D3BJ for the PCET 2*CO coupling and *COH formation reactions on the 2S slabs in continuum water (ε = 78.4) using Φ_{solv}= 0.373 and 0.403 eV, respectively.

Reaction	Φ [eV]			$\frac{(\Phi_a+\Phi_b)}{2}$ [eV]	
	R	T	P	R,T	R,P
$2{}^*CO + Cu_4 + e^- \rightarrow [OC^*–{}^*CO]^{(1+\delta)-} + Cu_4^{\delta+}$	3.429	3.824	4.109	3.627	3.769
$2{}^*CO + Cu_4 + e^- + H^+ \rightarrow [HOC^*–{}^*CO]^{\delta-} + Cu_4^{\delta+}$	3.429	-	4.361	-	3.895
${}^*CO + e^- + H^+ \rightarrow {}^*COH$	4.081	4.669	4.693	4.375	4.387

Supplementary References

(1) Huang, C.; Pavone, M.; Carter, E. A. Quantum mechanical embedding theory based on a unique embedding potential. *Journal of Chemical Physics* **2011**, *134* (15), 154110.
(2) Wu, Q.; Yang, W. A Direct Optimization Method for Calculating Density Functionals and Exchange–Correlation Potentials from Electron Densities. *Journal of Chemical Physics* **2003**, *118*, 2498-2509.
(3) Yu, K.; Libisch, F.; Carter, E. A. Implementation of density functional embedding theory within the projector-augmented-wave method and applications to semiconductor defect states. *Journal of Chemical Physics* **2015**, *143* (10), 102806.
(4) Yu, K.; Libisch, F.; Schlipf, M.; Boyn, J.-N.; Martirez, J. M. P.; Carter, E. A. *VASPEmbedding*. 2024. https://github.com/EACcodes/VASPEmbedding (accessed 2024 October).
(5) Martirez, J. M. P.; Carter, E. A. Insights into non-electroactive C-C bond formation on Cu(100) during electrochemical CO_2 reduction from multiconfigurational wavefunction theory *submitted* **2025**.
(6) Wen, X.; Boyn, J.-N.; Martirez, J. M. P.; Zhao, Q.; Carter, E. A. Strategies to Obtain Reliable Energy Landscapes from Embedded Multireference Correlated Wavefunction Methods for Surface Reactions. *Journal of Chemical Theory and Computation* **2024**, *20* (14), 6037-6048.
(7) Ghigo, G.; Roos, B. O.; Malmqvist, P. A. A modified definition of the zeroth-order Hamiltonian in multiconfigurational perturbation theory (CASPT2). *Chemical Physics Letters* **2004**, *396* (1-3), 142-149, Article.
(8) Roos, B. O.; Andersson, K. Multiconfigurational perturbation-theory with level shift - the Cr2 potential revisited. *Chemical Physics Letters* **1995**, *245* (2-3), 215-223.
(9) Chan, K.; Nørskov, J. K. Electrochemical Barriers Made Simple. *The Journal of Physical Chemistry Letters* **2015**, *6* (14), 2663-2668.
(10) Trasatti, S. The absolute electrode potential: an explanatory note (Recommendations 1986). *Journal of Electroanalytical Chemistry and Interfacial Electrochemistry* **1986**, *209* (2), 417-428.
(11) Isse, A. A.; Gennaro, A. Absolute Potential of the Standard Hydrogen Electrode and the Problem of Interconversion of Potentials in Different Solvents. *The Journal of Physical Chemistry B* **2010**, *114* (23), 7894-7899.
(12) Bader, R. F. W. *Atoms in Molecules: A Quantum Theory*; Clarendon Press, 1994.
(13) Tang, W.; Sanville, E.; Henkelman, G. A grid-based Bader analysis algorithm without lattice bias. *Journal of Physics-Condensed Matter* **2009**, *21* (8), 084204.
(14) Zhao, Q.; Martirez, J. M. P.; Carter, E. A. Electrochemical Hydrogenation of CO on Cu(100): Insights from Accurate Multiconfigurational Wavefunction Methods. *Journal of Physical Chemistry Letters* **2022**, 10282-10290.

(15) Zhao, Q.; Martirez, J. M. P.; Carter, E. A. Revisiting Understanding of Electrochemical CO_2 Reduction on Cu(111): Competing Proton-Coupled Electron Transfer Reaction Mechanisms Revealed by Embedded Correlated Wavefunction Theory. *Journal of the American Chemical Society* **2021**, *143* (16), 6152-6164.
(16) Li, L.; Martirez, J. M. P.; Carter, E. A. Prediction of Highly Selective Electrocatalytic Nitrogen Reduction at Low Overpotential on a Mo-Doped g-GaN Monolayer. *ACS Catalysis* **2020**, *10* (21), 12841-12857.
(17) Henkelman, G.; Uberuaga, B. P.; Jonsson, H. A climbing image nudged elastic band method for finding saddle points and minimum energy paths. *Journal of Chemical Physics* **2000**, *113* (22), 9901-9904.
(18) Chase, M. W.; Davies, C. A.; Downey, J. R.; Frurip, D. J.; McDonald, R. A.; Syverud, A. N. *NIST JANAF Thermochemical Tables 1985*; National Institute of Standards and Technology, 1986.
